# Influence of sequence variation on the RNA cleavage activity of Zn^2+^-dimethyl-dppz-PNA-based artificial enzymes†

**DOI:** 10.1039/d1ra08319h

**Published:** 2022-02-14

**Authors:** Olivia Luige, Kristina Karalė, Partha Pratim Bose, Martin Bollmark, Ulf Tedebark, Merita Murtola, Roger Strömberg

**Affiliations:** Department of Biosciences and Nutrition, Karolinska Institutet Neo, 141 83 Huddinge Sweden roger.stromberg@ki.se; RISE, Department of Chemical Process and Pharmaceutical Development Forskargatan 18 15136 Södertälje Sweden

## Abstract

The development of Zn^2+^-dependent dimethyl-dppz-PNA conjugates (PNAzymes) as efficient site-specific artificial ribonucleases enables rapid sequence-specific degradation of clinically relevant RNA target sequences, but the significance of the RNA/PNAzyme sequence and structural demands for the identification of novel RNA targets are not fully understood. In the present study, we investigated the influence of sequence variation in the recognition arms of the RNA/PNAzyme complex on the RNA cleavage activity of the artificial enzymes. The base pairs closing the 3-nucleotide bulge region on both sides of the bulge as well as the neighbouring nucleobases were shown to significantly influence the RNA cleavage activity. Elongation of the RNA/PNAzyme complex was shown to be tolerated, although potentially prohibitive for catalytic turnover. The specificity of PNAzyme action was clearly demonstrated by the significantly reduced or absent cleavage activity in complexes containing mismatches. Further investigation into 2- and 4-nucleotide RNA bulges indicated that formation of 3-nucleotide bulges in the target RNA gives the optimal cleavage rates, while some potential off-target cleavage of formed 4-nucleotide bulges of select sequences should be considered.

## Abstract 2

The ability of Zn^2+^ dimethyl-dppz PNAzymes to cleave RNA target sequences with under 20 minute half-lives is critically dependent on the bulge-closing base pairs as well as their stacking interactions with the neighbouring nucleobases.

## Graphical abstract

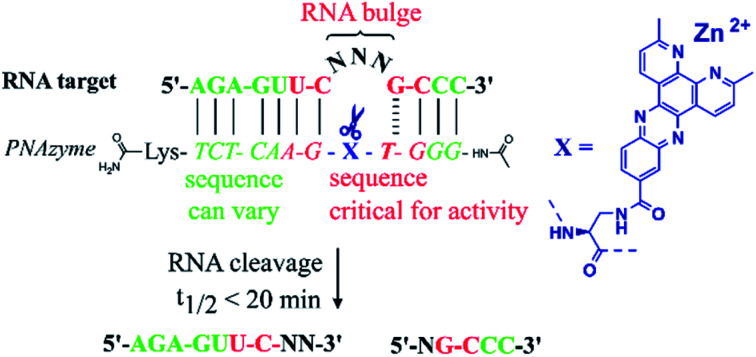

## Introduction

Nucleic acid manipulation is important in molecular biology^1^ as well as for therapeutic interventions in the clinic.^2,3^ The development of efficient artificial enzymes capable of sequence-specific cleavage of RNA has been a long-standing goal of nucleic acid chemistry.^4–8^ Such artificial ribonucleases have the potential to achieve degradation of disease-related RNA targets without the assistance of endogenous enzymes, thereby offering an alternative to gapmer antisense oligonucleotides (ASOs) and small interfering RNAs (siRNAs),^3,9^ both of which are used in the clinic today.

Artificial ribonucleases of various designs have been reported, including both metal-free^10–13^ and metal cofactor-dependent systems.^14–22^ The self-reliance of metal independent systems is attractive,^10^ although higher RNA cleavage rates have been accomplished with metal-dependent artificial nucleases.^16,18,19^ Zn^2+^ is an attractive cofactor due to its relative biocompatibility, and has been used in artificial ribonucleases developed with both 2′-*O*-methyl RNA^14,20,22^ and PNA backbones.^17,21^ However, Zn^2+^-dependent artificial ribonucleases have typically shown inferior RNA cleavage kinetics and lower site-specificty^14,17,20–22^ compared to Cu^2+^-dependent peptide nucleic acid–neocuproine conjugates (Cu^2+^-neocuproine PNAzymes).^16,18,19^ Nonetheless, the recently reported novel Zn^2+^-dependent dimethyl-dipyridophenazine (dppz)-based PNAzymes are a breakthrough, as they accomplish rapid RNA cleavage with down to 16 min half-lives at pH 7.0 and 10 min at pH 7.4.^15^ In addition, these artificial enzymes can be tailored to disease-related RNA targets.^15^ These PNAzymes are designed to be partially complementary to the RNA target sequences in order to force the formation of a bulge in the RNA adjacent to the dimethyl-dppz “molecular scissors”, thus taking advantage of the inherent reactivity of phosphodiester linkages within single-stranded RNA bulges.^23,24^ Fast site-specific cleavage was shown for 3-nucleotide bulge-forming RNAs, including clinically relevant RNA target sequences (*i.e.* a malaria parasite *Plasmodium falciparum* mRNA model target and a SARS-CoV-2 genomic RNA model target).^15^ While this significant development has successfully demonstrated rapid RNA cleavage of clinically relevant RNA models, the extent to which the target sequence can be modified while still maintaining efficient cleavage activity is still not fully understood. A thorough understanding of the intricacies of the sequence–activity relationships in the RNA/PNAzyme complexes can shed light on various aspects of PNAzyme action, while also being critical for the effective identification of novel RNA targets and for understanding possible off-target effects. The present study takes a particular interest in the bulge-closing base pairs on both sides of the bulge, as well as the sequence and length of the shorter recognition arm of the RNA/PNAzyme complex. Moreover, in addition to the cleavage of 3-nucleotide RNA bulges, which have been studied in detail,^15^ we demonstrate the cleavage of 2-nucleotide RNA bulges and study the sequence-dependence in 4-nucleotide bulges.

## Results and discussion

### Sequence–activity relationships in the long recognition arm

**Fig. 1:**
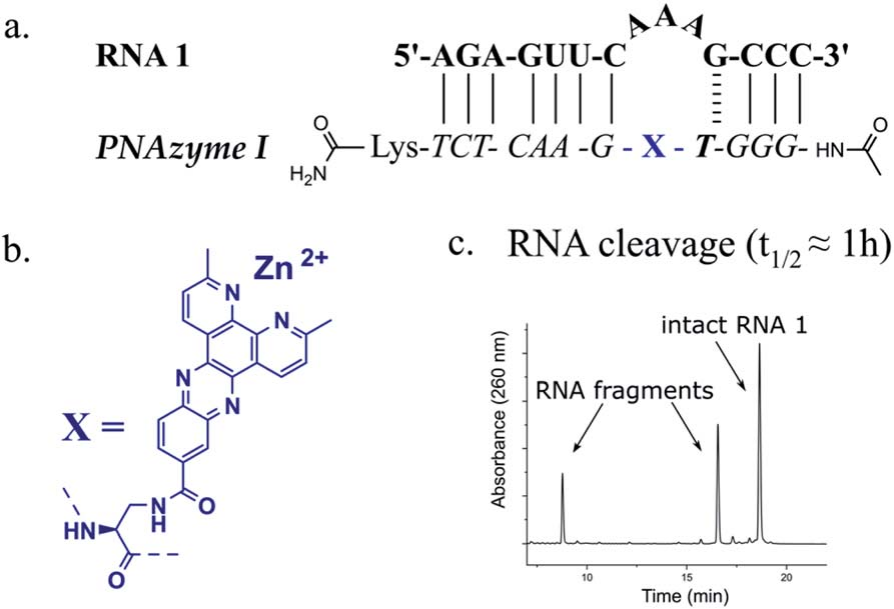
(a) Schematic representation of the previously reported RNA target 1 that forms a 3-nucleotide AAA bulge when in complex with PNAzyme I (b) structure of the dimethyl-dipyridophenazine (dppz) “molecular scissors” moiety which was conjugated to PNA to create Zn^2+^ dimethyl-dppz PNAzymes (c) previously reported (by Luige *et al.*)^15^ IEX-HPLC chromatogram showing the extent of cleavage of RNA 1 by Zn^2+^-dependent PNAzyme I after 50 min of incubation at 37 °C, pH 7 in the presence of 100 μM Zn^2+^.

**Fig. 2:**
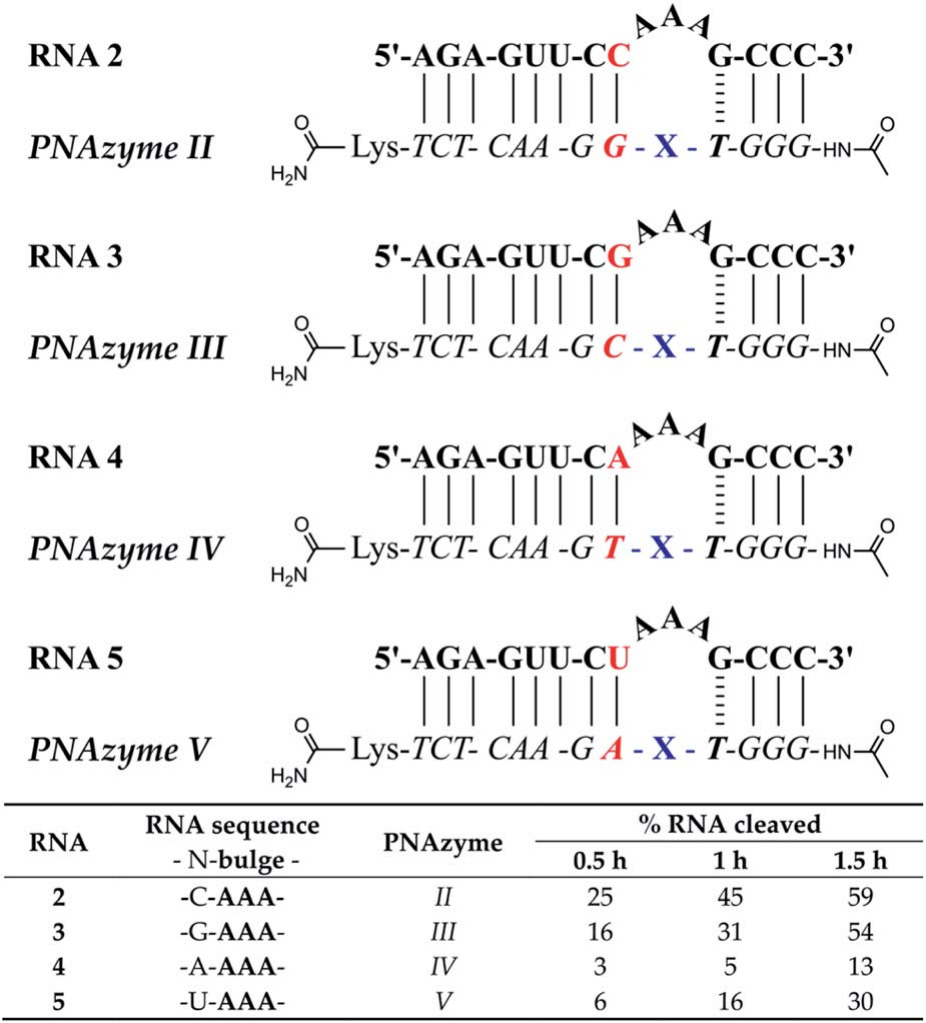
Schematic representations of RNA/PNAzyme complexes where the bulge-closing base pair in the long recognition arm varies, followed by the extent of RNA cleavage observed in each complex after incubation of the RNA/PNAzyme complex (4 μM) in the presence of Zn^2+^ (100 μM) at 37 °C, pH 7. The structure of “X” (the “molecular scissors”) is shown in Fig. 1b.

The reported RNA/PNAzyme complexes (Fig. 1) contain a CG base pair closing the bulge in the longer hybridised stem, *i.e.* the long recognition arm.^15^ In such systems, the AAA bulge is cleaved site-specifically with an approximately 1 h half-life (Fig. 1c).^15^ Extension of the long recognition arm by one base pair gives essentially the same cleavage rate (Fig. 2, RNA 2 and PNAzyme II). In order to further investigate the role of the bulge-closing base pair in the long recognition arm, we constructed novel systems where variations were introduced to the RNA 2/PNAzyme II sequence (Fig. 2). Four PNA-dimethyl-dppz conjugates were compared (PNAzymes II–V, Fig. 2), where AAA bulges are formed in the corresponding RNA targets (RNAs 2–5, Fig. 2), while the bulge-closing base pair is varied in each complex.

The cleavage of RNA 2, where the bulge was closed with a CG pair, gave the highest cleavage rate while the reversed GC bulge-closing base pair (RNA 3/PNAzyme III) showed slightly lower activity. Rather strikingly, the cleavage activity suffered a major loss when the AAA bulge was closed with a UA (RNA 5/PNAzyme V) or AT base pair (RNA 4/PNAzyme IV), where the RNA cleavage half-lives were estimated to be in the 4 and 11 h range, respectively (Fig. 2). MS analysis confirmed that the cleavage site was between the second and third nucleotide in the bulge (5′-AA/A-3′), while the least efficiently cleaved RNA 4 also showed significant cleavage between the third bulge nucleotide and the GT wobble (5′-AAA/G-3′) (see ESI-S3† for IE-HPLC chromatograms and MS data).

The overall dependence of RNA cleavage activity on the bulge-closing base pairs (most to least favourable CG > GC > UA > AT) suggests that the strength of hydrogen bonding is key. Moreover, there seems to be an additional benefit if the pyrimidine is in the RNA and the purine in the PNA strand. Herein as well as in prior reports,^15^ the nucleobase preceding the bulge-closing base pair has been either of the two pyrimidine bases in the RNA and the complementary purine in the PNA strand. As such, it should be carefully considered in the selection of novel RNA targets in future investigations whether instead of UC or CC (pyrimidine-C), perhaps AG or GG (purine-G) sequence would be tolerated equally well in the bulge-closing region of the long recognition arm. It should be noted that the activity of more complex triplex-forming Cu^2+^-neocuproine PNAzymes has indeed been higher with purine–purine (AG) rather than purine-pyrimidine (AC) sequence in the RNA target in the same bulge-closing region.^18^

The key role of the bulge-closing base pair suggests it has a profound influence on the structure of the bulge. Indeed, bulges are known to be diverse structural motifs with nucleotide arrangements defined by the competing interactions of the bulge nucleotides and the surrounding hybridised base pairs.^25^ The bulge nucleobases can point out into the solvent, fold back onto adjacent hydrophobic surfaces, or they may take part in the continuous stacking of the surrounding double strands, favouring kinking of the helix.^25^ As such, the bulge-closing base pair is clearly changing the structural arrangement, which is also evident in the comparison of the circular dichroism (CD) spectra of these four complexes (see ESI-S4†).

**Fig. 3:**
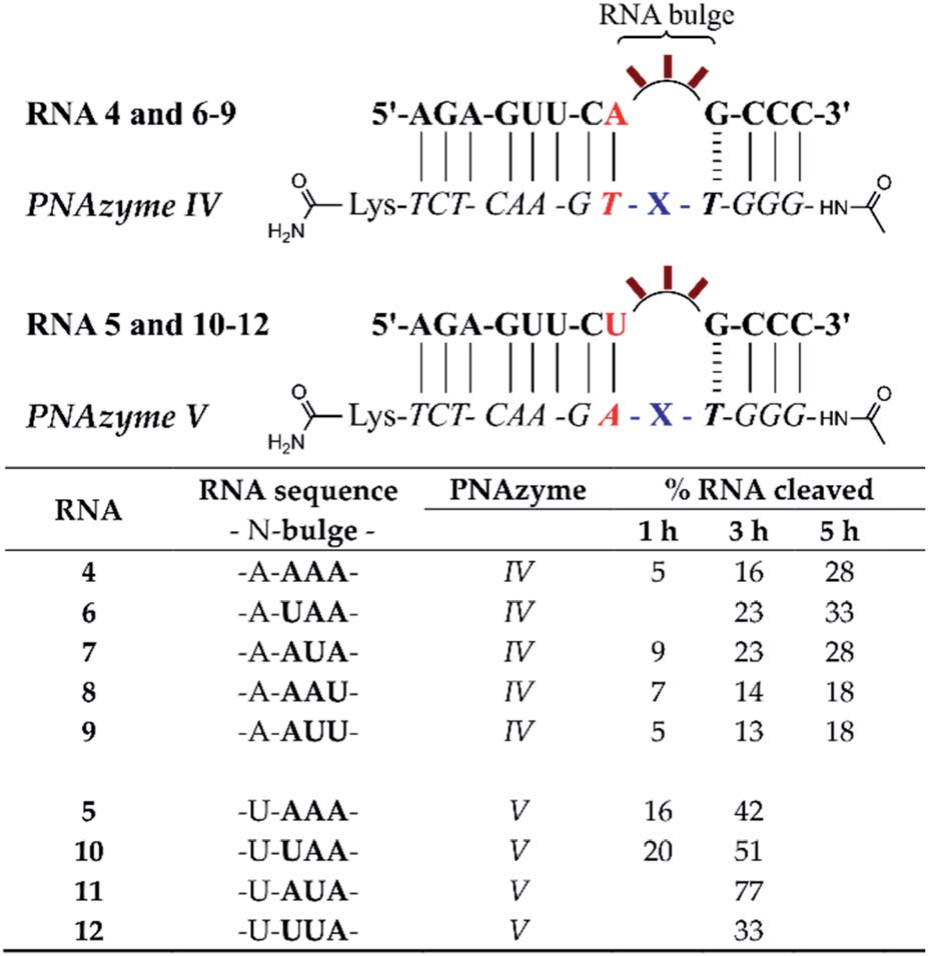
Schematic representations of RNA/PNAzyme complexes where the 3-nucleotide RNA bulge sequence is varied in complexes with either an AT or UA bulge-closing base pair in the long recognition arm, followed by the extent of RNA cleavage observed in each complex after incubation of the RNA/PNAzyme complex (4 μM) in the presence of Zn^2+^ (100 μM) at 37 °C, pH 7. The structure of “X” (the “molecular scissors”) is shown in Fig. 1b.

Generally, PNAzyme-promoted cleavage of RNA has been shown to exhibit characteristic bulge-sequence dependence.^15–17,19^ For RNA/Zn^2+^-dimethyl-dppz PNAzyme complexes where the recognition arms have the sequence shown for RNA 1/PNAzyme I in Fig. 1, changing the RNA bulge sequence from AAA (Fig. 1) to AUA, GUA or UUA enhances the cleavage rate, corresponding to a shift in the half-life from 1 h to 16 min.^15^ In order to determine whether similar sequence dependence could occur in the less efficient systems where the bulge is closed with an AT or UA base pair in the long recognition arm, we investigated RNA targets with other bulge sequences in these systems. Interestingly, complexes with the AT pair did not exhibit the characteristic bulge sequence-dependence to a significant extent. Some variation was observed in the extent of cleavage (with adenosine being favoured over uridine in the third position), but the activity remained very low for all five bulge sequences (RNAs 4, 6–9, Fig. 3 and ESI-S3†). Nonetheless, complexes with the UA bulge-closing base pair displayed some bulge sequence-dependence (RNAs 5 and 10–12, Fig. 3 and ESI-S3†). The fastest rate was observed for the AUA bulge (RNA 11), where the cleavage took place at a single site (5′-AU/A-3′, see ESI-S3†) with an estimated half-life in the 1.5 h range, which is an improvement from the approximately 4 h half-life reported above for the AAA bulge closed with the same base pair. However, the overall cleavage activity of the systems where 3-nucleotide bulges were closed with AT or UA base pairs was significantly lower than the previously reported 16 min half-lives for the systems with a CG bulge-closing base pair.^15^

**Fig. 4:**
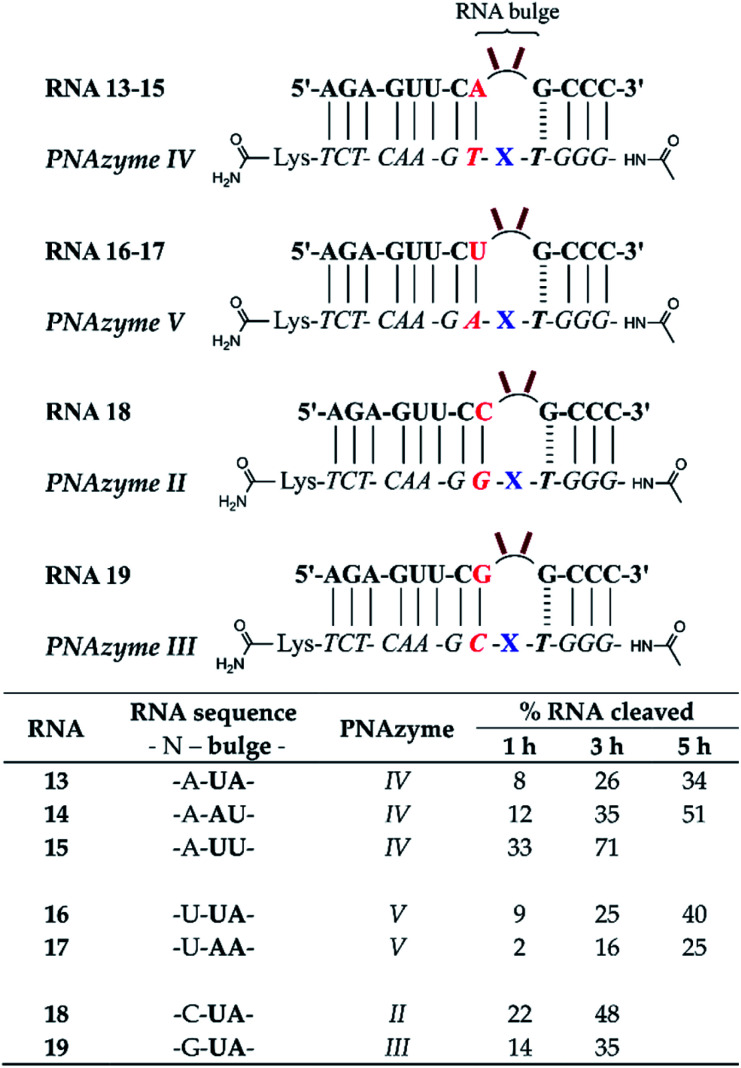
Schematic representations of RNA/PNAzyme complexes where 2-nucleotide RNA bulges are formed, followed by the extent of RNA cleavage observed in each complex after incubation of the RNA/PNAzyme complex (4 μM) in the presence of Zn^2+^ (100 μM) at 37 °C, pH 7. The structure of “X” (the “molecular scissors”) is shown in Fig. 1b.

To further probe the activity of complexes with the weaker bulge-closing base pairs, we investigated whether faster cleavage rates could be accomplished by targeting smaller 2-nucleotide bulges (Fig. 4). Notably, a significantly improved cleavage rate was indeed observed for the AT bulge-closing base pair when the RNA formed a UU bulge (RNA 15). In this case the cleavage was site-specific (5′-UU/G-3′, see ESI-S3†) with an estimated half-life in the 1.5 h range.

Comparison of the cleavage of the 2-nucleotide UA bulges closed with different base pairs showed that the RNA/PNAzyme complexes which contained the CG and GC bulge-closing base pairs (RNAs 18 and 19) once again outperformed those with the AT and UA pairs (RNAs 13 and 16, Fig. 4), although their activity was significantly higher with 3-nucleotide bulges. In the case of all 2-nucleotide UA bulge-forming systems regardless of the bulge-closing base pair, cleavage fragments originating from two cleavage sites (5′-U/A-3′ and 5′-UA/G-3′) were detected (see ESI-S3†). These results suggest that 3-nucleotide RNA bulges closed with CG or GC base pairs in the long recognition arm are the optimal targets since these systems give the highest overall cleavage rates. Moreover, the sequence dependence in 2-nucleotide bulges closed with a CG or GC base pair could be a subject for future investigations.

### Sequence–activity relationships in the short recognition arm

**Fig. 5:**
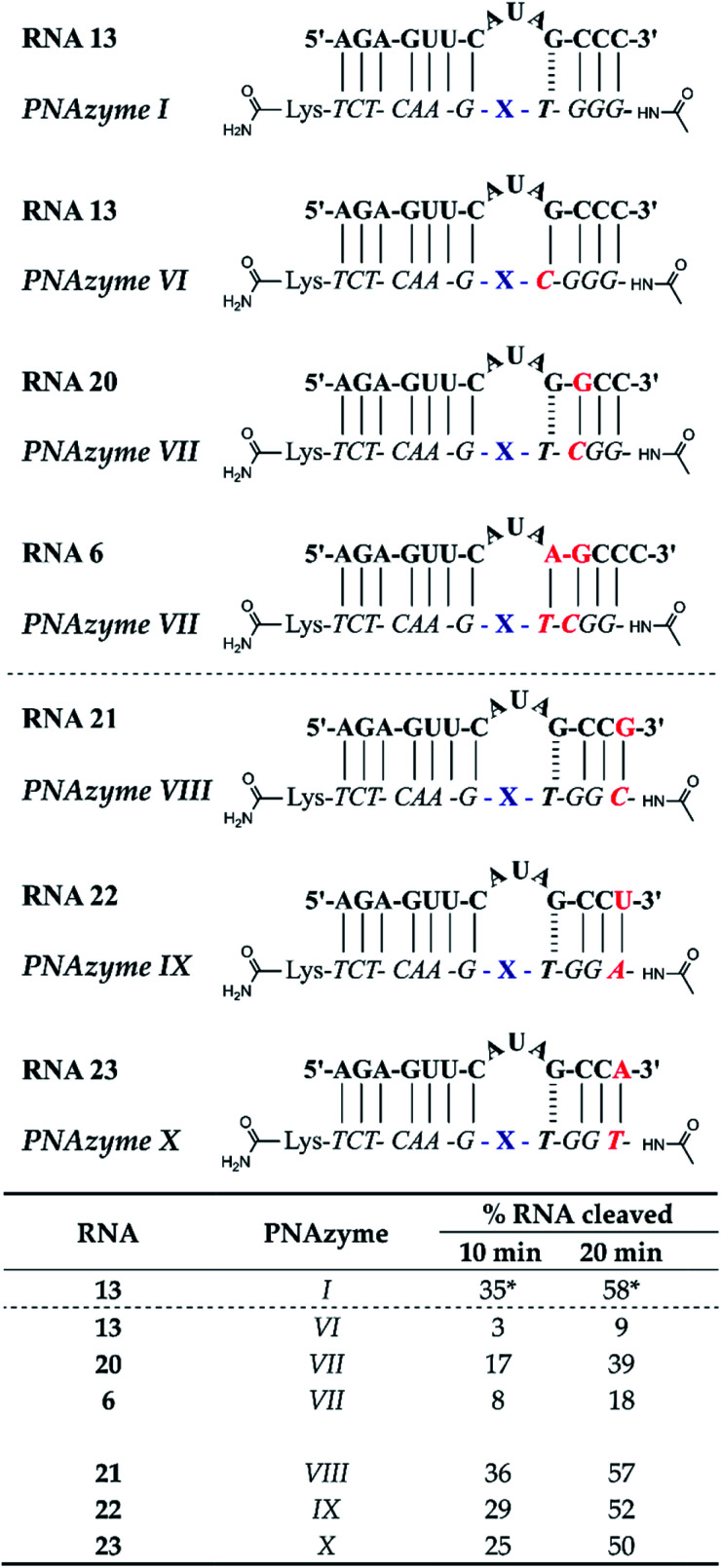
Schematic representations of RNA/PNAzyme complexes where variations are introduced in the short recognition arm, followed by the extent of RNA cleavage observed in each complex after incubation of the RNA/PNAzyme complex (4 μM) in the presence of Zn^2+^ (100 μM) at 37 °C, pH 7. The structure of “X” (the “molecular scissors”) is shown in Fig. 1b. *The extent of cleavage of RNA 13 by PNAzyme I has been previously reported by Luige *et al.*^15^

The short recognition arm of the RNA/PNAzyme complex comprises a GT wobble, followed by three CG Watson–Crick base pairs. The replacement of the GT wobble with a GC Watson–Crick base pair in Cu^2+^-neocuproine PNAzymes decreased the RNA cleavage activity substantially.^19^ Here, we synthesised a Zn^2+^-dimethyl dppz PNAzyme designed to form a GC base pair instead of the GT wobble with RNA 13 (Fig. 5). The replacement of the GT wobble with a GC base pair (RNA 13/PNAzyme VI) significantly lowered the RNA cleavage rate (Fig. 5). A profound structural change was also evident in the comparison of the CD spectra of these complexes (ESI-S4†).

The position next to the bulge-closing base pair in the short recognition arm was then modified to include a GC instead of the CG base pair next to the GT wobble (RNA 20/PNAzyme VII). Interestingly, this change in the position next to the bulge-closing base pair in the short recognition arm led to a slightly lower cleavage rate. As such, the GC sequence in RNA 13 closing the bulge in the short recognition arm was favoured over GG in RNA 20. It remains unclear whether the strength of hydrogen bonding in the position next to the bulge-closing base pair is critical, or perhaps GU (purine–pyrimidine) would be tolerated equally well, hence allowing the possibility of a UA base pair in the RNA/PNAzyme complex next to the bulge-closing GT wobble.

Moreover, a further change was then introduced by replacing the GT wobble with an AT bulge-closing base pair, which was then followed by a GC base pair (RNA 6/PNAzyme VII). A further decrease in the RNA cleavage rate was observed. The cleavage still took place at a single site between the second and third nucleotide in the bulge (5′-AU/A-3′; see ESI-S3†).

In summary, the bulge-closing base pair in the short recognition arm had an influential role on the RNA cleavage activity. The GT wobble was clearly preferred, while the AT base pair was shown to be detrimental, yet tolerated more than a GC base pair.

The significant effects of altering the sequence in the immediate vicinity of the bulge led us to investigate the influence of the terminal base pair in the short recognition arm. We synthesised PNAzymes VIII–X (Fig. 5), which preserve the first three base pairs in the short recognition arm but form different terminal base pairs with RNAs 21–23. In summary the alteration of the terminal base gave almost the same cleavage rates as with the CG pair in RNA13/PNAzyme I, which perhaps is not unexpected since terminal base pairs are known to be more flexible.

### Elongation of the RNA/PNAzyme complex and catalytic turnover

**Fig. 6:**
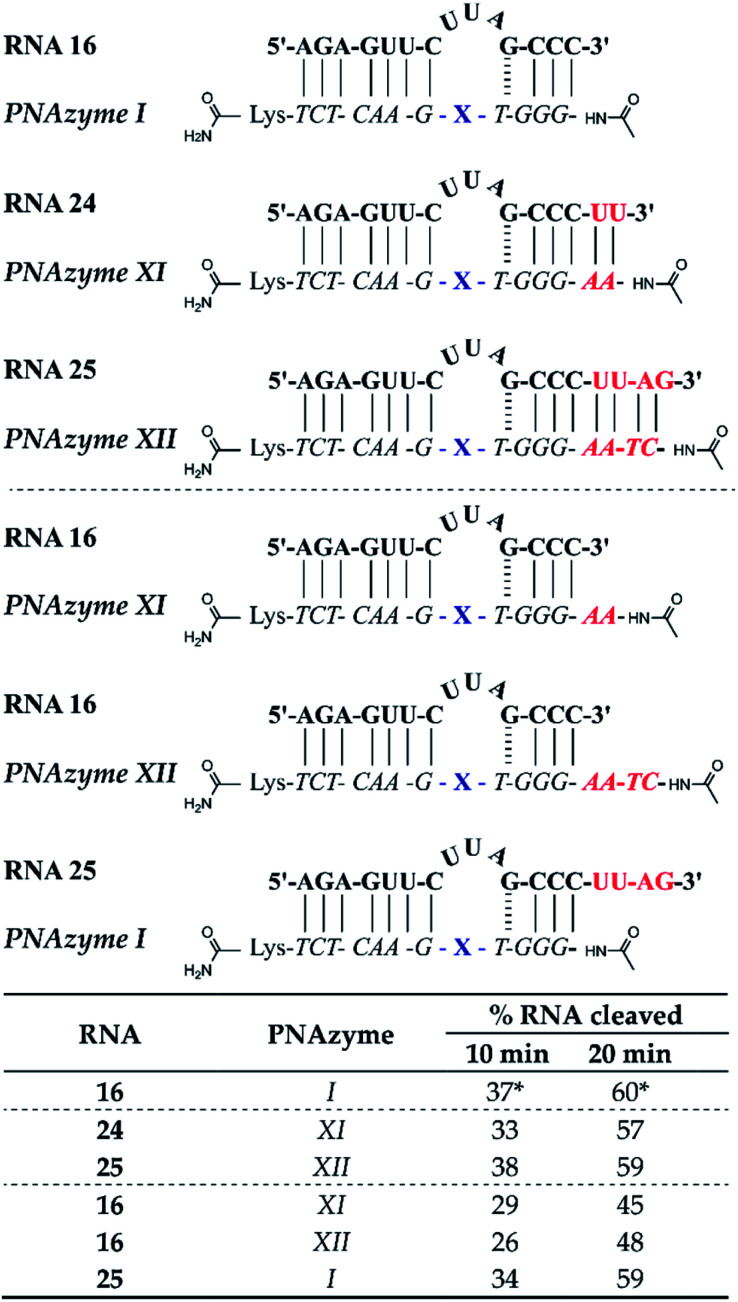
Schematic representations of elongated RNA/PNAzyme complexes where the standard short recognition arm (comprising 3 Watson–Crick and 1 wobble base-pair) is extended by 2 and 4 Watson–Crick base pairs, as well as the extension of only the PNA strand which creates an overhang with the standard RNA target, followed by the extent of RNA cleavage observed in each complex after incubation of the RNA/PNAzyme complex (4 μM) in the presence of Zn^2+^ (100 μM) at 37 °C, pH 7. The structure of “X” (the “molecular scissors”) is shown in Fig. 1b. *The extent of cleavage of RNA 16 by PNAzyme I has been previously reported by Luige *et al.*^15^

Apart from altering the sequence of the recognition arms in the RNA/PNAzyme complex, elongation of the complex is another alteration that could potentially have implications for the cleavage efficacy. Additionally, if extension of the sequence is tolerated, then this may aid in structural studies (*e.g.* crystallography with a non-cleavable modification at the cleavage site) since the complex stability will be higher. The previously studied complexes (*e.g.* RNA 1/PNAzyme I) consist of two recognition arms comprising 7 Watson–Crick base pairs on the one side and 1 GT wobble followed by 3 Watson–Crick base pairs on the other side. The elongated complexes studied herein contained elongated short recognition arms. The RNA 24/PNAzyme XI and RNA 25/PNAzyme XII complexes have two and four additional Watson–Crick base pairs at the RNA 3′ terminus. Elongation of the complex in the short recognition arm led to virtually the same extent of cleavage as the shorter system in the presence of equimolar PNAzyme (Fig. 6). Before this experiment we reasoned that the base pairs possibly need to “breathe” in order for the cleavage to take place, which could explain the critical role of the weak GT wobble closing the bulge. The fact that the extended stem systems are as efficient suggests that this is not needed. Moreover, elongation of the complex may be beneficial, as it can increase the specificity of PNAzyme binding.

In addition, we investigated the cleavage of complexes where either the PNAzyme or the RNA target sequence had an overhang (Fig. 6). The elongated PNAzyme overhang had a minor detrimental effect on the cleavage rate of RNA 16 both in the case of PNAzyme XI and XII. The significance of these results is especially relevant in the context of possible off-target sequences, where the recognition arm is incomplete, thus resulting in a PNAzyme overhang. Although it is likely that such off-targets would contain other mismatches that would potentially eliminate any catalytic activity, it is interesting that even just a short PNA overhang can decrease cleavage efficiency.

The RNA overhang, on the other hand, was shown not to affect the rate of cleavage of RNA 25 by PNAzyme I. This is an encouraging indication for cleavage of longer RNA sequences that would be the targets in potential therapeutic applications. This is also consistent with previous studies that demonstrated that Cu^2+^-neocuproine PNAzymes readily cleave elongated RNAs that are more than double the length of the PNAzyme.^16^ An additional point of concern could be interference from other biologically significant metals, however, Na, Mg and K were shown not to interfere with Zn^2+^-PNAzyme-mediated RNA cleavage (see ESI-S5†).

**Fig. 7:**
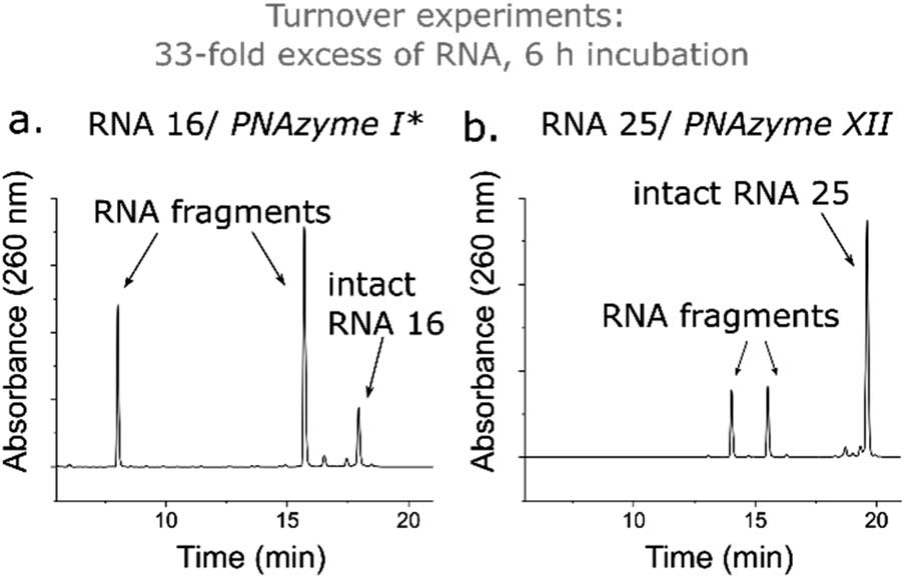
IEX-HPLC chromatograms showing the extent of cleavage of a 33-fold excess of (a) RNA 16 (14mer) and (b) RNA 25 (18mer) (100 μM) by their corresponding PNAzymes (3 μM) after 6 h of incubation in the presence of Zn^2+^ (100 μM) at 37 °C, pH 7. *The data shown in panel (a) has been previously reported by Luige *et al.*^15^

While the rate of cleavage is unaffected by elongation of the short recognition arm, the length of the complex can be more critical for catalytic turnover, *i.e.* for the ability of each PNAzyme molecule to cleave multiple RNA targets. In order to achieve efficient turnover, the cleaved fragments must readily dissociate from the PNAzyme, so that the PNAzyme can bind to the next intact RNA target. The short RNA 16/PNAzyme I complex has been shown to give turnover.^15^ The elongated RNA 25/PNAzyme XII system studied herein gave 34 ± 2% RNA cleavage in 6 h under turnover conditions (33-fold excess of RNA compared to the PNAzyme, Fig. 7b). While multiple turnover was clearly demonstrated, the extent of cleavage was somewhat lower than the previously reported 86 ± 4% for the short complex under the same conditions (Fig. 7a).^15^ An explanation could be that elongation of the short recognition arm beyond 7 Watson–Crick base pairs decreases the rate of dissociation of the cleaved fragments, thus limiting turnover. Furthermore, these results suggest that further extension of the 7 Watson–Crick base pairs (which include 3 CG or GC pairs) in the long recognition arm would likely also adversely affect turnover.

### Bulge sequence-dependence in the cleavage of 4-nucleotide RNA bulges

Prior reports have indicated that the cleavage of the AAAA bulge can be several times slower than the AAA bulge.^15^ While the latter is evidently a better target, the possibility of off-target cleavage of the former should be considered. Moreover, the rate of cleavage of 3-nucleotide bulges is greatly increased when the AAA bulge is replaced with UUA, AUA or GUA bulges (from *ca.* 1 h half-life to *ca.* 16 min at pH 7).^15^ It is likely that the sequence of the 4-nucleotide bulges would also have a significant influence on the cleavage rate, but this sequence-dependence requires further investigation.

**Fig. 8:**
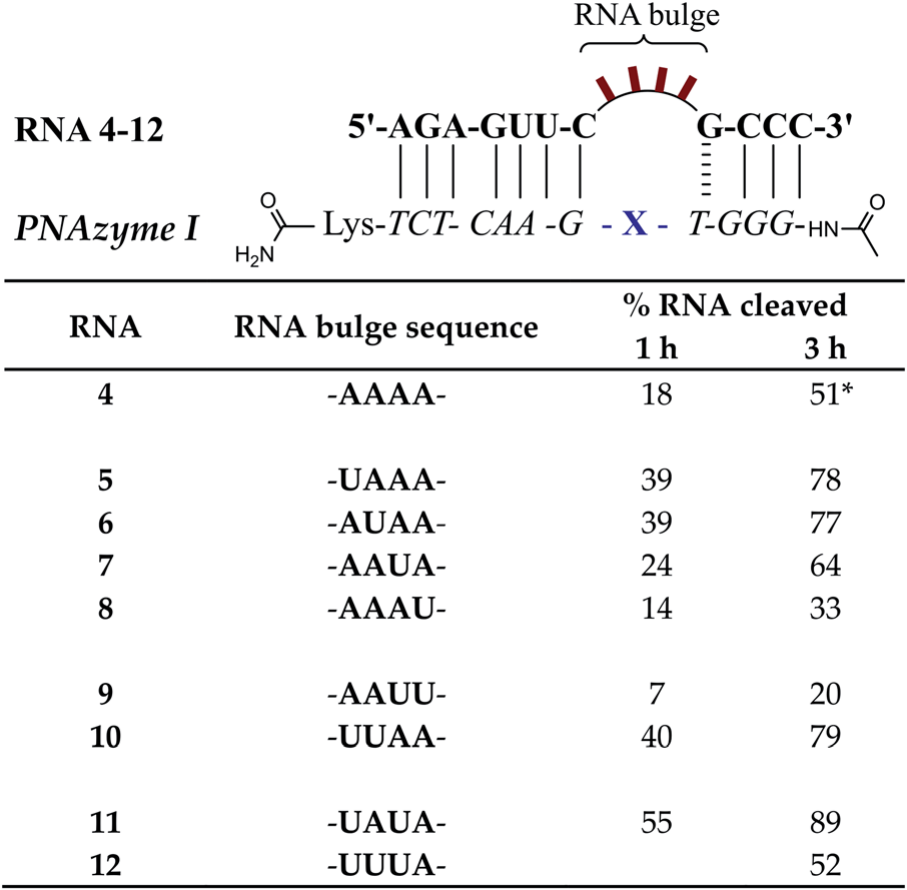
Schematic representations of RNA/PNAzyme complexes where 4-nucleotide RNA bulges are formed, followed by the extent of RNA cleavage observed in each complex after incubation of the RNA/PNAzyme complex (4 μM) in the presence of Zn^2+^ (100 μM) at 37 °C, pH 7. The structure of “X” (the “molecular scissors”) is shown in Fig. 1b. *The extent of cleavage of RNA 4 by PNAzyme I in 3 h has been previously reported by Luige *et al.*^15^

In the present report, RNAs 5–12 which form 4-nucleotide bulges of various sequences composed of adenosine and uridine in different positions were studied and compared to the cleavage of the AAAA bulge-forming RNA 4 (see Fig. 8). A significant increase in the cleavage rate was observed for RNA bulges where uridine was introduced in the first or second position (RNAs 5–6) or simultaneously in both positions (UUAA, RNA 10). The cleavage rate was also slightly increased when uridine was present in the third position (AAUA, RNA 7). In fact, the fastest cleavage rate was achieved when adenosine was replaced with uridine simultaneously in the first and third position (UAUA bulge, RNA 11) in which case the estimated cleavage half-life was below 1 h and the cleavage occurred in a nearly site-specific fashion. The predominant cleavage site in RNA 11 was between the third and fourth bulge nucleotide (5′-UAU/A-3′, see ESI-S3†), in contrast to RNA 4 where cleavage fragments resulting from two equally favoured cleavage sites (AA/AA and 5′-AAA/A-3′) were detected. While the presence of one or two uridines in the first three positions enhanced the cleavage rate, interestingly, the presence of three uridines in these positions (UUUA bulge in RNA 12) did not appear to affect the cleavage rate compared to the AAAA bulge in RNA 4. Similarly to the previously reported sequence dependence in 3-nucleotide bulges,^15^ the preservation of the preferred 5′-U/A-3′ or A/A cleavage site was clearly important also in 4-nucleotide bulges, as the presence of uridine in the position next to the GT wobble (AAAU bulge in RNA 8) led to a slight decrease in the cleavage rate and two consecutive uridines next to the GT wobble (AAUU bulge, RNA 9) were even more detrimental to the cleavage rate (Fig. 8).

**Fig. 9:**
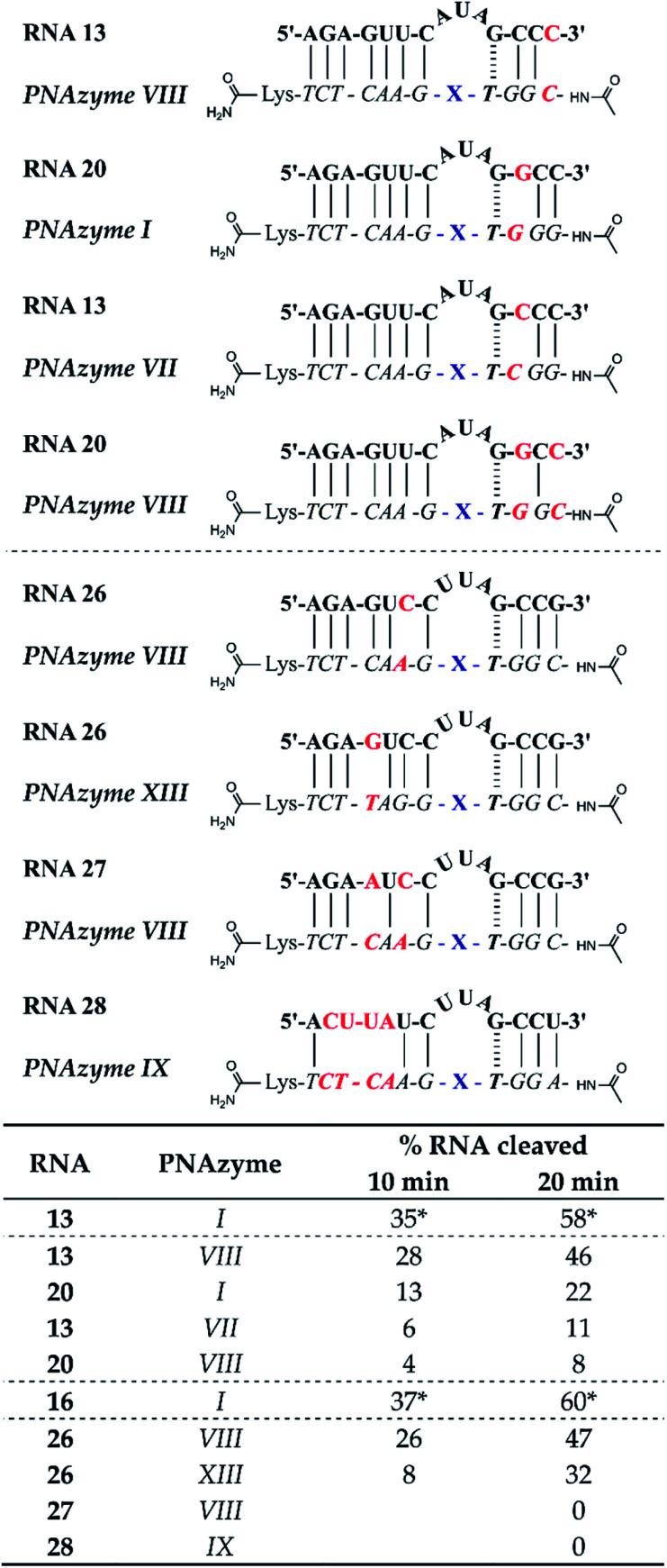
Schematic representations of RNA/PNAzyme complexes containing various mismatches, followed by the extent of RNA cleavage observed in each complex after incubation of the RNA/PNAzyme complex (4 μM) in the presence of Zn^2+^ (100 μM) at 37 °C, pH 7. The structure of “X” (the “molecular scissors”) is shown in Fig. 1b. *The extent of cleavage of RNAs 13 and 16 by PNAzyme I has been previously reported by Luige *et al.*^15^

### Mismatch intolerance in RNA/PNAzyme complexes

Lastly, we investigated the cleavage of mismatched RNA sequences (Fig. 9) to gain a deeper understanding of the specificity of PNAzymes. A terminal mismatch had only a slight negative effect on the cleavage rate, while a single mismatch in the central position in the short recognition arm or two mismatches simultaneously at the central and terminal position reduced the extent of RNA cleavage considerably. Rather interestingly, a mismatch in the long recognition arm in the position adjacent to the bulge-closing base pair had only a modest negative effect, while a GT wobble in the central part of the long recognition arm reduced the cleavage rate somewhat more. The presence of two or more mismatches in the long recognition arm completely abolished the catalytic activity. In summary, the sequence-specific nature of PNAzyme action was clearly reflected in the results. Although some cross reactivity can occur with highly similar sequences, mismatches in the RNA/PNAzyme complexes are clearly discriminative. Taken together with the requirements for the bulge sequence, the results imply high selectivity in target RNA cleavage.

## Conclusions

This study has shown that the RNA cleavage activity is critically dependent on the sequence of the RNA/PNAzyme complex. The cleavage of the AAA bulge occurred with estimated half-lives ranging from approximately 1–1.5 h to 4 h and 11 h, depending on whether the bulge was closed with a CG, GC, UA or AT base pair in the long recognition arm, respectively. Moreover, 2-nucleotide bulges of the same sequence (UA) were cleaved more efficiently when the complex contained the CG and GC bulge-closing base pairs instead of UA and AT pairs. Thus, the bulge-closing base pair was suggested to have a critical role in defining the structural arrangement of the bulge nucleotides, while interactions involving the base pair preceding the bulge-closing base pair are also likely to be important.

The bulge-closing base pair in the short recognition arm was equally influential, as the highest rates were obtained with a GT wobble, and both AT and GC Watson–Crick base pairs had a negative effect on the cleavage rate. The activity was also affected by the succeeding base pair.

These sequence–activity relationships shed light on the structural demands for efficient cleavage of RNA targets by the Zn^2+^-dimethyl-dppz-PNAzymes. For rapid site-specific cleavage, the RNA targets are suggested to require the sequence 5′-Py-C-bulge-G-C-3′ (where Py = pyrimidine, and the bulge sequence can be UUA, AUA or GUA), although the feasibility of the RNA sequence Pu-G (Pu = purine) instead of Py-C preceding the bulge and G-U following the bulge could also be evaluated. Variations in the rest of the sequence are likely to be tolerated. Furthermore, the RNA/PNAzyme complex can be longer than the standard 11 base pairs, albeit the catalytic turnover might be affected if either of the cleaved fragments forms more than 7 base pairs with the PNAzyme. Consequently, these structural demands must be taken into consideration in the selection of RNA target sequences for PNAzymes in future studies. Although RNA targets forming 3-nucleotide bulges are clearly more efficiently cleaved, potential off-target sequence cleavage should be considered, as the PNAzymes were shown to also cleave 4-nucleotide RNA bulges of select sequences with down to 1 h half-lives.

## Experimental

All reagents and solvents used were of analytical commercial quality. RNA cleavage experiments were performed in nuclease-free water for molecular biology purchased from Sigma-Aldrich (St. Louis, Missouri, United States). Oligoribonucleotides RNA 1–23 were purchased from Dharmacon (Lafayette, CO, USA). RNA 1 and 13 were purchased purified. RNAs 2–12 and 14–23 were purified as previously reported.^15^ Oligoribonucleotides RNA 26–28 were purchased purified from integrated DNA Technologies, Inc (Coralville, Iowa, USA). RNA 13, 16, 24 and 25 were synthesised as previously reported.^15^ Concentrations of RNA sequences were determined by UV absorption at 260 nm on a Varian Cary 300 UV-Vis dual beam spectrophotometer (Varian, Palo Alto, CA, USA) using extinction coefficients obtained by the nearest neighbour approximation.^26^

Rink Amide resin (ChemMatrix, 0.47 mmol g^−1^) was purchased from Biotage (Uppsala, Sweden). Peptide nucleic acid monomers, Fmoc-PNA-A(Bhoc)-OH, Fmoc-PNA-G(Bhoc)-OH, Fmoc-PNA-C(Bhoc)-OH and Fmoc-PNA-T-OH, were purchased from Link Technologies Ltd (Glasgow, UK). Fmoc-l-Dap(Mtt)-OH (2,3-diaminopropionic acid), Fmoc-l-Lys(Boc)-OH, *N*,*N*′-diisopropylcarbodiimide (DIC) and 1-[bis(dimethylamino)methylene]-1*H*-1,2,3-triazolo[4,5-*b*]pyridinium 3-oxide hexafluorophosphate (HATU) were purchased from Iris Biotech GmbH (Marktredwitz, Germany). Ethyl cyano(hydroxyimino) acetate (Oxyma) was purchased from Merck-Millipore (Burlington, MA, USA). Acetic anhydride and 2,6-lutidine were purchased from Alfa Aesar (Haverhill, Massachusetts, United States). *N*-Methylpyrrolidone (NMP), dichloromethane (DCM), triisopropylsilane (TIS), piperidine and *N*-methylmorpholine (NMM) were purchased from Sigma-Aldrich (St. Louis, Missouri, USA). Trifluoroacetic acid (TFA) was purchased from Fisher Scientific (Pittsburgh, Pennsylvania, USA). Neocuproine and 3,4-diaminobenzoic acid were purchased from Fluorochem (Hadfield, UK).

### Preparation of PNAzymes I–XII

PNA oligomers were prepared on a Biotage Initiator+ Alstra microwave peptide synthesiser as previously reported.^15^ 3,6-Dimethyl-dipyrido[3,2-*a*:2′,3′-*c*]phenazine-11-carboxylic acid was synthesised as previously reported.^15^ PNAzyme I has been previously reported.^15^ PNA conjugates (PNAzymes II–XII) were prepared as previously reported.^15^ The RP-HPLC purity analysis of purified PNA conjugates is available in ESI-S1.† The purified PNA conjugates were identified by mass spectrometry on a Bruker Ultraflex MALDI-TOF mass spectrometer in positive ion mode using a sinapic acid matrix (10 mg mL^−1^ sinapic acid in acetonitrile–water (1 : 1, v/v) containing 0.1% TFA).

MALDI-MS *m/z*: PNAzyme I C_151_H_181_N_73_O_38_ [M + H]^+1^ calculated 3625.34, observed 3625.36. PNAzyme II C_162_H_194_N_80_O_41_ [M + H]^+1^ calculated 3916.56, observed 3916.92. PNAzyme III C_161_H_194_N_78_O_41_ [M + H]^+1^ calculated 3876.56, observed 3877.13. PNAzyme IV C_162_H_195_N_77_O_42_ [M + H]^+1^ calculated 3891.56, observed 3891.73. PNAzyme V C_162_H_194_N_80_O_40_ [M + H]^+1^ calculated 3900.57, observed 3901.06. PNAzyme VI C_150_H_180_N_74_O_37_ [M + H]^+1^ calculated 3610.46, observed 3611.66. PNAzyme VII C_150_H_181_N_71_O_38_ [M + H]^+1^ calculated 3585.45, observed 3585.60. PNAzyme VIII C_150_H_181_N_71_O_38_ [M + H]^+1^ calculated 3585.45, observed 3585.29. PNAzyme IX C_151_H_181_N_73_O_37_ [M + H]^+1^ calculated 3609.46, observed 3609.30. PNAzyme X C_151_H_182_N_70_O_39_ [M + H]^+1^ calculated 3600.45, observed 3600.53. PNAzyme XI C_173_H_207_N_87_O_42_ [M + H]^+1^ calculated 4175.68, observed 4176.44. PNAzyme XII C_194_H_234_N_96_O_49_ [M + H]^+1^ calculated 4692.88, observed 4693.48.

Concentrations of PNA conjugates were determined by UV absorption at 260 nm on a Varian Cary 300 UV-Vis dual beam spectrophotometer (Varian, Palo Alto, CA, USA), using extinction coefficients obtained by the nearest neighbour approximation,^26^ and the reported extinction coefficient for dipyridophenazine.^27^

### RNA cleavage experiments

RNA cleavage experiments were performed and analysed as previously reported,^15^ but for clarity we requote them in ESI-S2.† Representative IEX-HPLC chromatograms showing the extent of RNA cleavage at specified timepoints are shown in ESI-S3.† RNA cleavage half-lives were estimated from the percentage of RNA cleavage at the given time points using the rate law for first order reactions since our experiments are performed under saturation conditions with respect to PNAzyme and Zn^2+^ ion binding.

### Determination of RNA cleavage sites

RNA cleavage experiments were performed as detailed above, but with a 22 hour incubation time. The cleaved fragments were then identified by LC-MS analysis of the quenched reaction aliquots as follows. HRMS spectra were collected by elution of the aliquots of RNA cleavage reactions on an Oligonucleotide BEH C18 (2.1 × 50 mm, 130 Å, 3 μm) column using a 0.4 mL min^−1^ linear gradient from 0 to 80% acetonitrile in 8.6 mM TEA + 100 mM HFIP buffer over 15 min at 40 °C on Waters Xevo G2-XS QTof. Ionization mode: ESI negative. Source capillary voltage: 2 kV. Source desolvation temperature: 320 °C. Full scan with fragmentation, mass range: 400–2000 Da/0.5 s, sensitivity mode. Deconvoluted using 5 ppm tolerance. The MS spectra of the cleaved fragments are shown in ESI-S3.

### Circular dichroism (CD) spectroscopy

CD spectra of RNA/PNAzyme complexes were measured between 230 and 370 nm on a Jasco J-1500 CD Spectrometer using 10 mm path length cuvettes. The spectra were recorded as an average of five scans at 25 °C and normalised by subtracting the background buffer scans. The samples of RNA/PNAzyme complexes (4 μM) were analysed in the absence of Zn^2+^ ions in 10 mM phosphate buffer containing 100 mM NaCl and 0.1 mM EDTA at pH 7.0. The spectra were smoothed over 5 points. The spectra are shown in ESI-S4.†

## Author contributions

Conceptualisation – O. L., M. M. and R. S. Formal analysis – O. L. and R. S. Funding acquisition – R. S. Investigation – O. L and K. K. with contributions from P. B., M. B. Methodology – O. L., M. M., R. S. Project administration – O. L., R. S. Resources – U. T., R. S. Supervision – M. B., U. T., R. S. Visualisation – O. L. Writing - original draft – O. L. Writing – review and editing – O. L. and R. S. with contributions from all.

## Disclosures

Since performing this work, Olivia Luige has become an employee of AstraZeneca.

## Conflicts of interest

There are no conflicts to declare.

## Supplementary Material

RA-012-D1RA08319H-s001
