# Supplementary material for: Influence of sequence variation on the RNA cleavage activity of Zn2+-dimethyl-dppz-PNA-based artificial enzymes
Source: RSC Adv. 2022 Feb 14;12(9):5398–406. doi: 10.1039/d1ra08319h (PMC8981518; doi:10.1039/d1ra08319h)
Supplement: RA-012-D1RA08319H-s001 [file RA-012-D1RA08319H-s001.pdf]

Electronic Supplementary Material (ESI) for RSC Advances.
This journal is © The Royal Society of Chemistry 2022

Influence of sequence variation on the RNA cleavage activity of Zn^{2+}-dimethyl-dppz-PNA-based artificial enzymes

Olivia Luige,[a] Kristina Karalė,[ab] Partha Pratim Bose,[a] Martin Bollmark,[b] Ulf Tedebark,[b] Merita Murtola [a] and Roger Strömberg *[a]

a Department of Biosciences and Nutrition, Karolinska Institutet, Neo, 141 83 Huddinge, Sweden.
b RISE Chemical Process and Pharmaceutical Development, Forskargatan 18, 15136 Södertälje, Sweden

Electronic Supplementary Information

S-1. Purity analysis of PNA conjugates 2
S-2. RNA cleavage experiments 8
S-3. Analysis of RNA Cleavage by IEX-HPLC and ESI-TOF MS 9
S-3.1 The extent of cleavage of AAA bulges in RNAs 2-5 by the corresponding *PNAzymes II-V* 9
S-3.2 MS analysis of RNA fragments from the cleavage of RNAs 2-4 by *PNAzymes II-IV* 10
S-3.3 The extent of cleavage of 3-nucleotide bulge-forming RNAs 6-9 by *PNAzyme IV* 12
S-3.4. The extent of cleavage of 3-nucleotide bulge-forming RNAs 10-12 by *PNAzyme V* 14
S-3.5. MS analysis of RNA fragments from the cleavage of RNA 11 by *PNAzyme V* 15
S-3.6. The extent of cleavage of 2-nucleotide bulge-forming RNAs 13-15 by *PNAzyme IV* 15
S-3.7. MS analysis of RNA fragments from the cleavage of RNAs 13, 15 by *PNAzyme IV* 16
S-3.8. The extent of cleavage of 2-nucleotide bulge-forming RNAs 16-17 by *PNAzyme V* 18
S-3.9. MS analysis of RNA fragments from the cleavage of RNA 17 by *PNAzyme V* 18
S-3.10 The extent of cleavage of UA bulges in RNAs 18-19 by *PNAzymes II-III* 20
S-3.11. MS analysis of RNA fragments from the cleavage of RNA 18 by *PNAzyme II* 20
S-3.12. MS analysis of RNA fragments from the cleavage of RNA 19 by *PNAzyme III* 21
S-3.13 The extent of cleavage of RNA 6 by *PNAzyme VII* 22
S-3.14. MS analysis of RNA fragments from the cleavage of RNA 6 by *PNAzyme VII* 23
S-3.15 The extent of cleavage of 4-nucleotide bulge-forming RNAs 4-12 by *PNAzyme I* 24
S-3.16. MS analysis of RNA fragments from the cleavage of RNAs 4 and 11 by *PNAzyme I* 26
S-4. Circular Dichroism Spectroscopy of RNA/PNAzyme complexes 29
S-4.1. Complexes where the bulge-closing base pair varies in the long recognition arm 29
S-4.2 Complexes with a GT wobble vs a GC Watson-Crick base pair closing the bulge in the short recognition arm 29
S-5. RNA cleavage experiments in the presence of Mg^{2+} and K^+ 30
References 31

S-1. Purity analysis of PNA conjugates

The PNA conjugate PNAzyme I has been previously described and the RP-HPLC chromatograms of the crude and pure conjugate have been published.[1] The retention time of unconjugated PNA is around 15 min.[1]

Representative RP-HPLC chromatogram of the crude PNAzyme II:

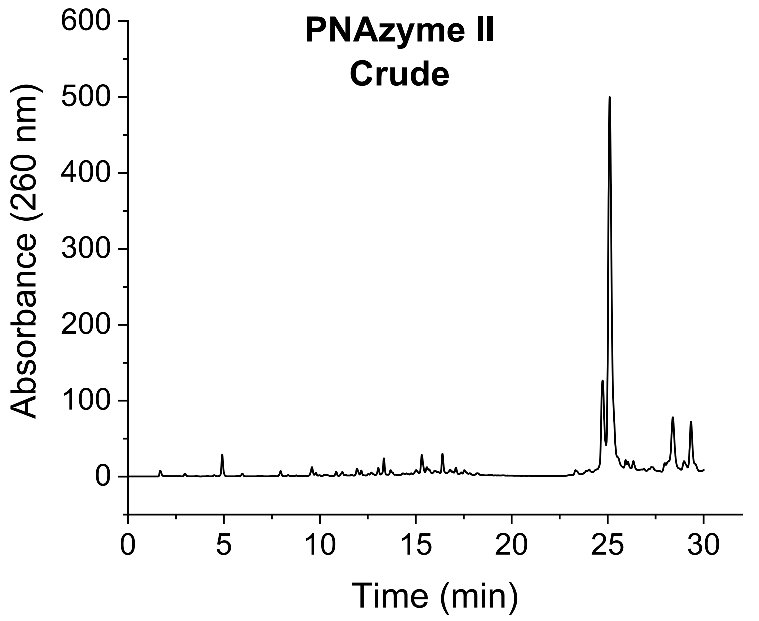

Purity analysis of purified PNA conjugates (PNAzymes II-XII):

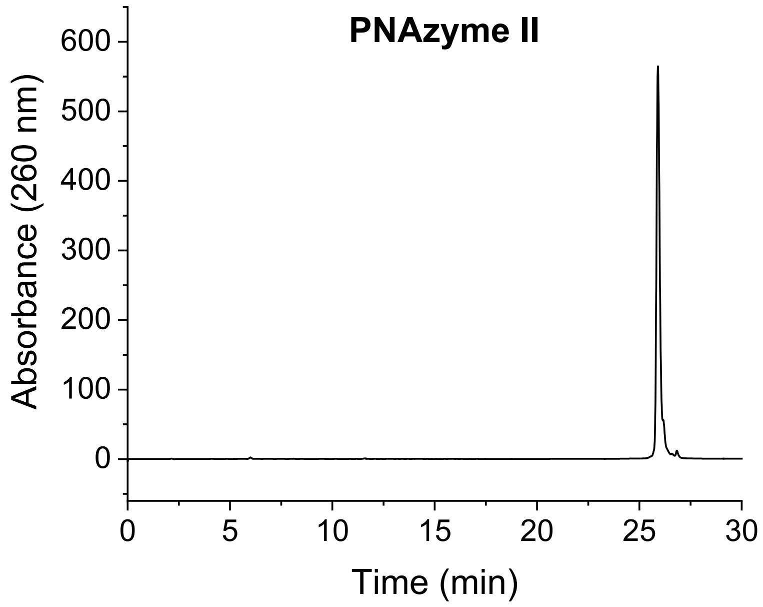

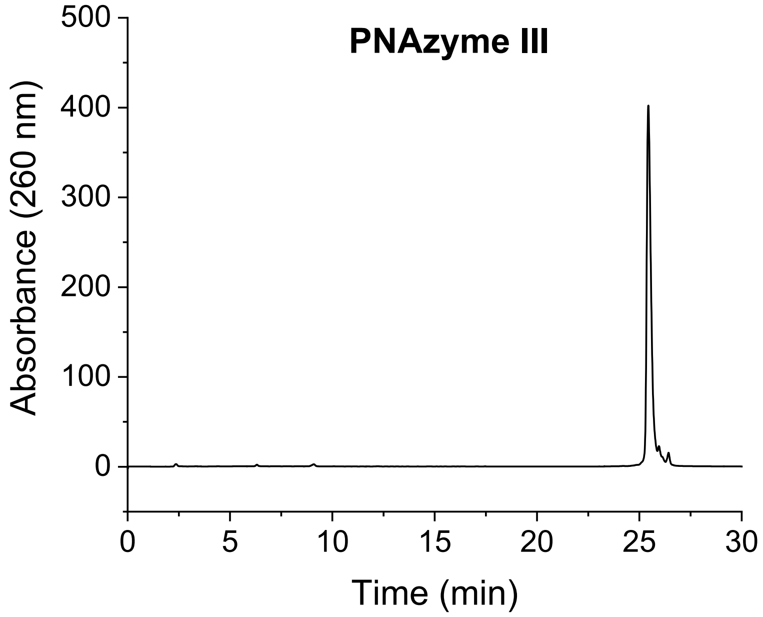
PNAzyme III
Absorbance (260 nm)
Time (min)
0
100
200
300
400
500
0
5
10
15
20
25
30

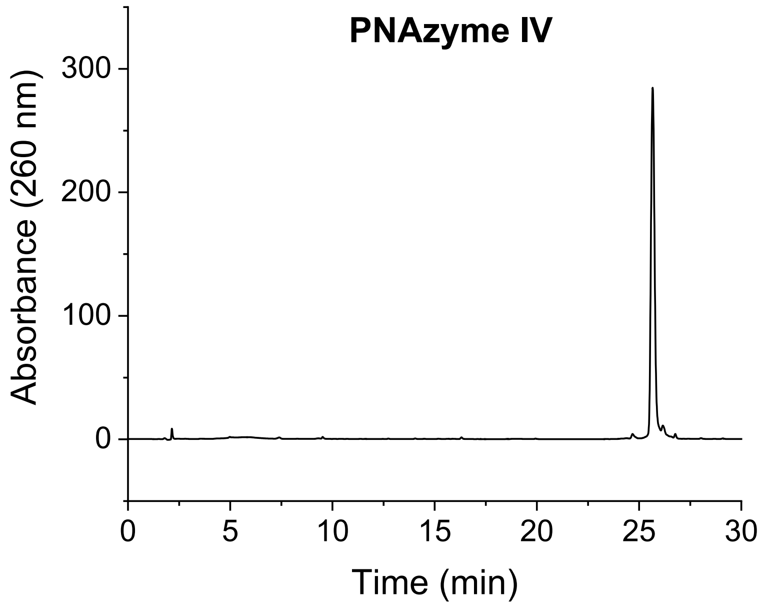
PNAzyme IV
Absorbance (260 nm)
Time (min)
0
100
200
300
0
5
10
15
20
25
30

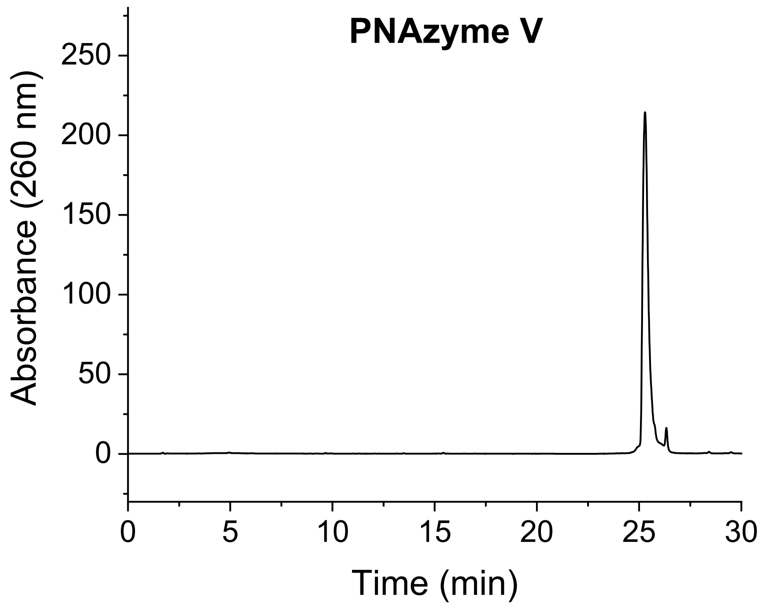
PNAzyme V
Absorbance (260 nm)
0
50
100
150
200
250
0
5
10
15
20
25
30
Time (min)

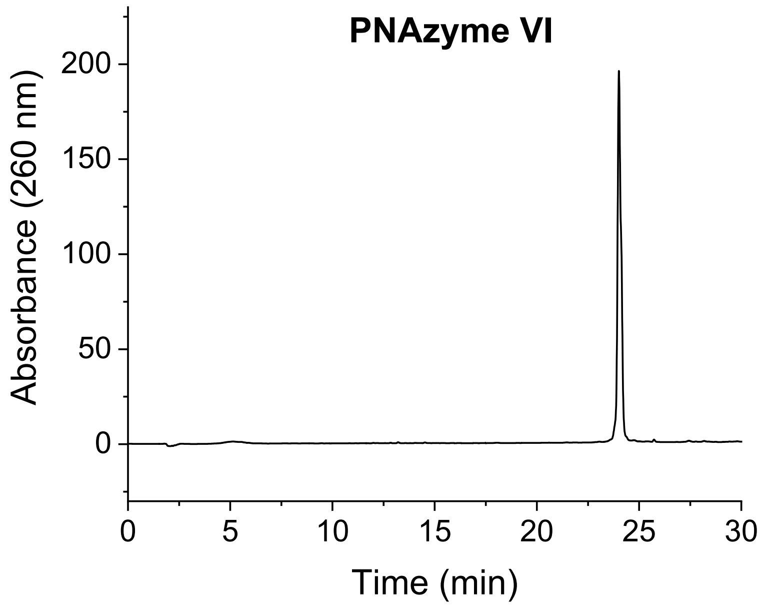
PNAzyme VI
Absorbance (260 nm)
0
50
100
150
200
0
5
10
15
20
25
30
Time (min)

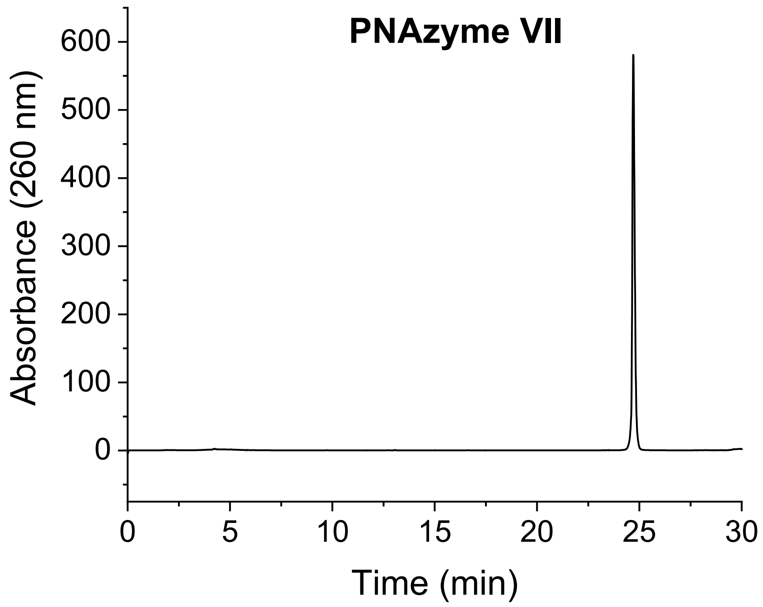
PNAzyme VII
Absorbance (260 nm)
600
500
400
300
200
100
0
0
5
10
15
20
25
30
Time (min)

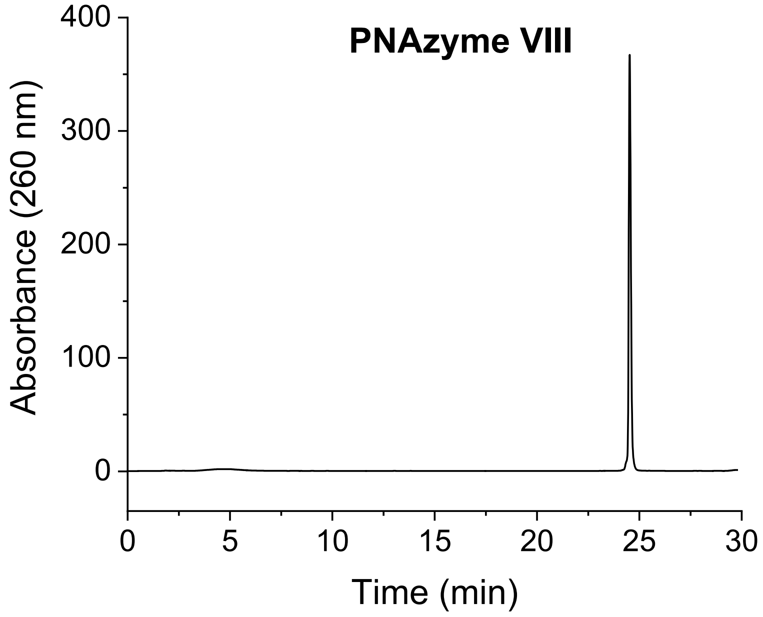
PNAzyme VIII
Absorbance (260 nm)
400
300
200
100
0
0
5
10
15
20
25
30
Time (min)

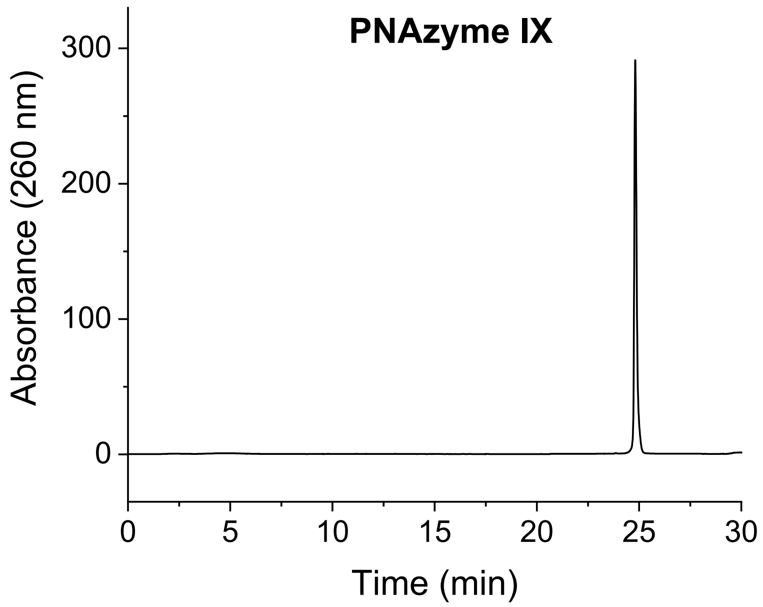
PNAzyme IX
Absorbance (260 nm)
300
200
100
0
0
5
10
15
20
25
30
Time (min)

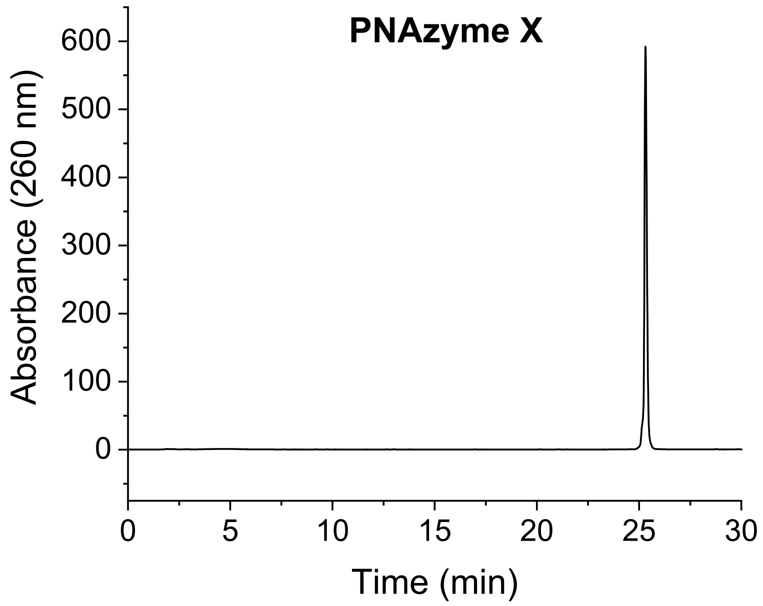
PNAzyme X
Absorbance (260 nm)
600
500
400
300
200
100
0
0
5
10
15
20
25
30
Time (min)

Representative RP-HPLC chromatogram of the crude PNAzyme XI:

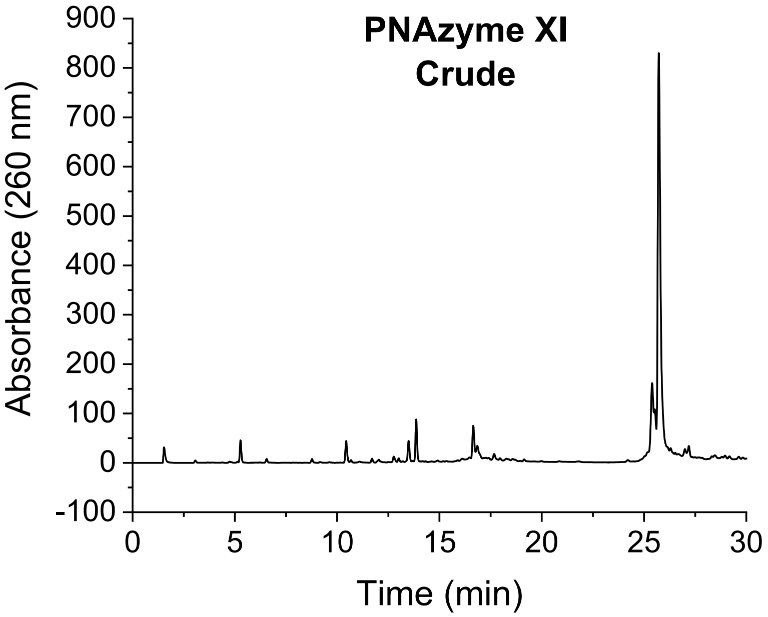

Purity analysis of purified PNAzymes XI and XII was performed using an extended method (0 to 33% buffer B in 40 min as opposed to the standard method[1] where the gradient is 0 to 40% buffer B in 30 min) leading to an increased retention time (~39-40 min).

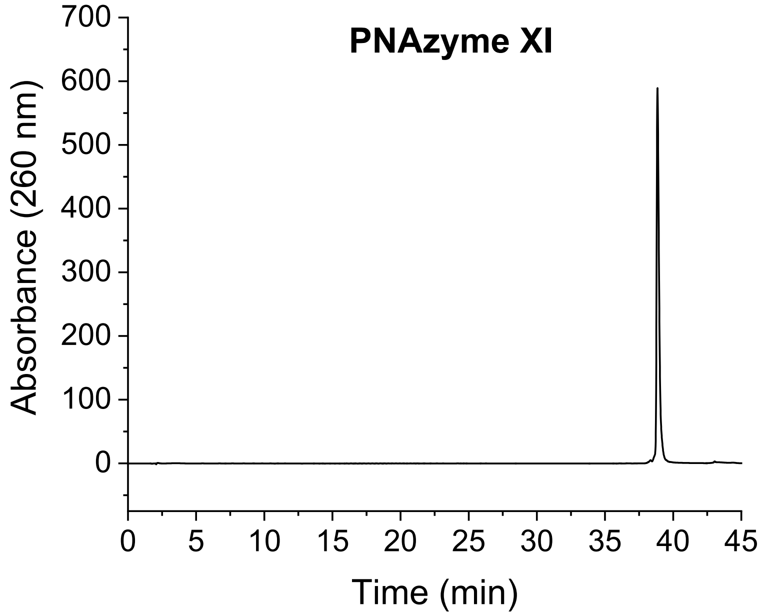

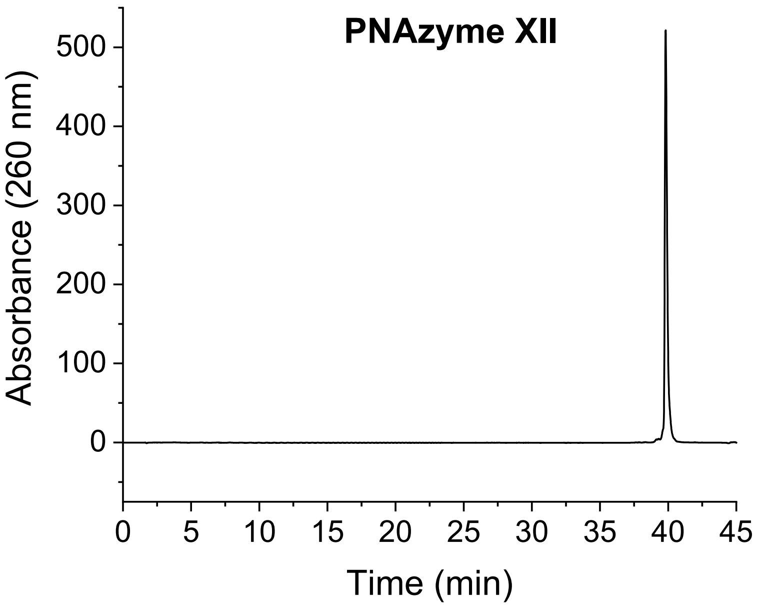

S-2. RNA cleavage experiments

RNA cleavage experiments were performed and analysed as previously reported,[1] but for clarity we requote it here. RNA targets (0.16 nmol, 4 µM final concentration, except in turnover experiments where the RNA concentration was 100 µM RNA) were equilibrated in appropriate amounts of water, HEPES buffer (10 mM HEPES, 0.1 M NaCl final concentration) and EDTA (10 µM final concentration) over a 15-minute period at 37 °C. This was followed by the addition of $Zn(NO_3)_2$ (*aq*) solution (110 µM Zn^{2+} which in the presence of the previously added 10 µM EDTA results in 100 µM accessible zinc ions), the PNAzyme (1.4 equiv relative to RNA, 5.6 µM, except in turnover experiments where the PNAzyme concentration was 3 µM). The reaction mixtures were then allowed to incubate at 37 °C. The reactions were quenched at specified time points with EDTA (70 µL of 2 mM EDTA in 30% MeCN/milliQ). The samples were analysed by anion exchange HPLC (IE-HPLC) using a Dionex NucleoPac PA-100 (4 × 250 mm) column with a linear gradient elution of 0–45% buffer B over 30 min at 60 °C. A flow rate of 1.5 mL/min was used and UV detection was carried out at 260 nm. The following solvent system was used: (A) 20 mM NaOAc in 30% aq. MeCN and (B) 20 mM NaOAc, 0.4 M $LiClO_4$ in 30% aq. MeCN. The extent of cleavage of RNA substrates was based on the quantification of the remaining intact RNA target and the sum of the formed RNA fragments detected in the IE-HPLC analysis. Representative chromatograms are provided. The % RNA cleavage values presented are average values of at least two experiments (except in turnover experiments where the reported values are the mean cleavage of at least five experiments) with a standard deviation of less than ± 3% unless otherwise specified.

S-3. Analysis of RNA Cleavage by IEX-HPLC and ESI-TOF MS

S-3.1 The extent of cleavage of AAA bulges in RNAs 2-5 by the corresponding *PNAzymes II-V*

Timepoints at which the aliquots were quenched are shown on each chromatogram. These data are shown in Fig 2 and 3 in the main text.

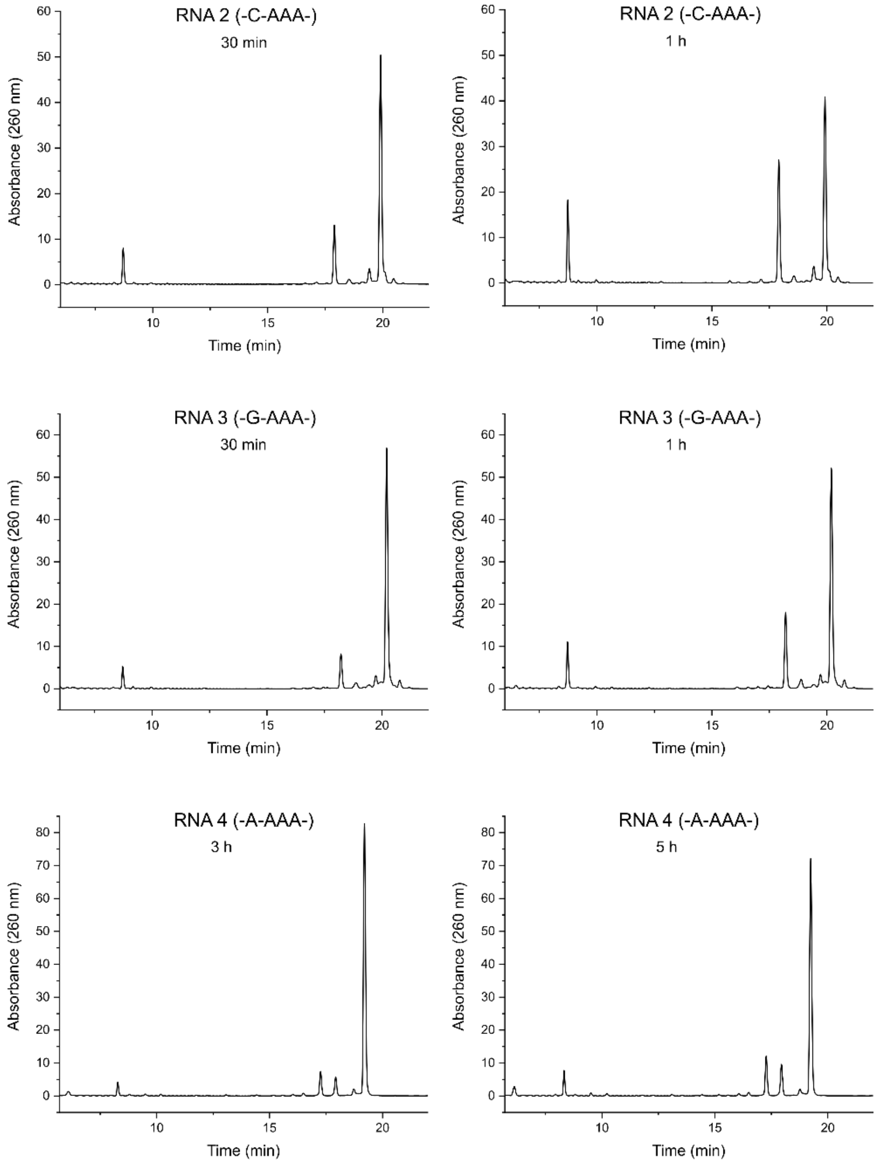

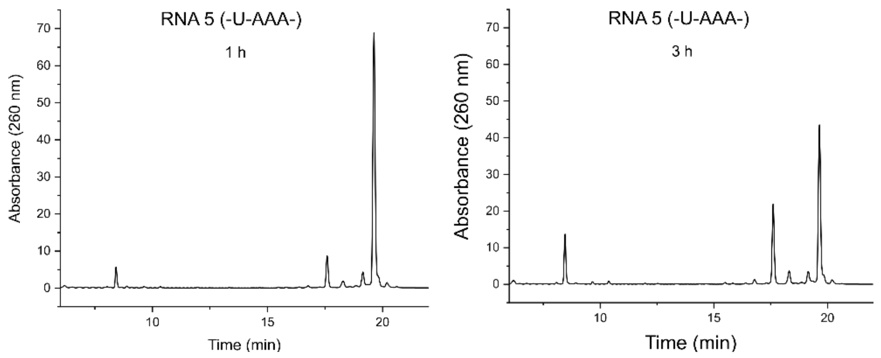

S-3.2 MS analysis of RNA fragments from the cleavage of RNAs 2-4 by *PNAzymes II-IV*

RNA 2 cleavage fragment 1 (5'-AGCCC-3') $C_{47}H_{61}N_{19}O_{32}P_4$
Monoisotopic mass calc. 1527.268, obs. 1526.285.

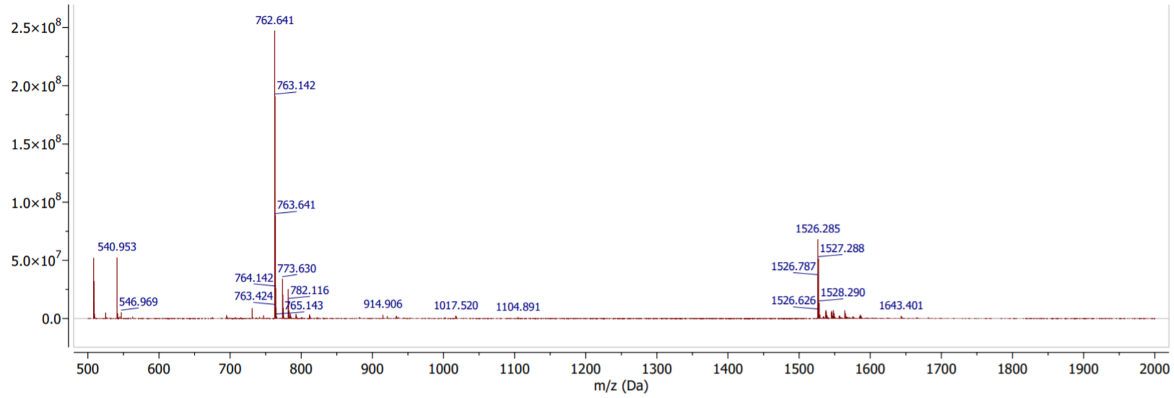

RNA 2 cleavage fragment 2 (2',3'-cyclic phosphate, 5'-AGAGUUC-C-AA-3') $C_{96}H_{118}N_{40}O_{68}P_{10}$
Monoisotopic mass calc. 3228.438, obs. 3228.495.

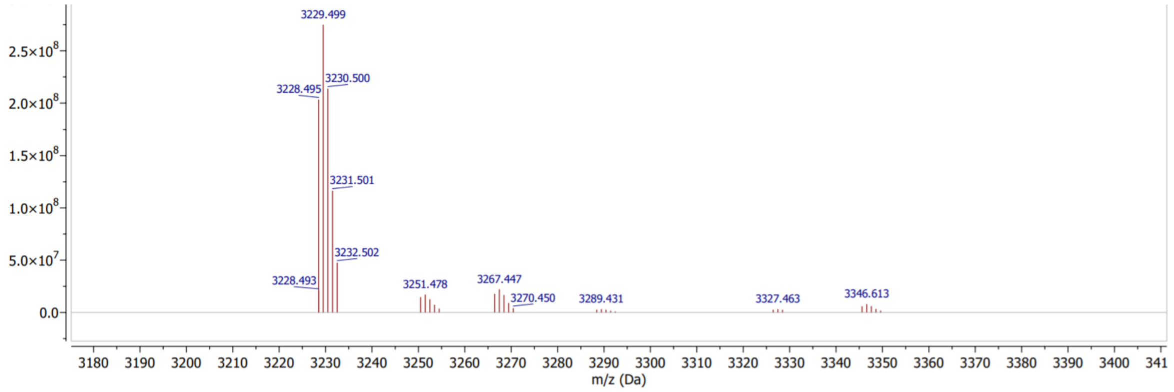

RNA 3 cleavage fragment 1 (5'-AGCCC-3') $C_{47}H_{61}N_{19}O_{32}P_4$
Monoisotopic mass calc. 1527.268, obs. 1526.285.

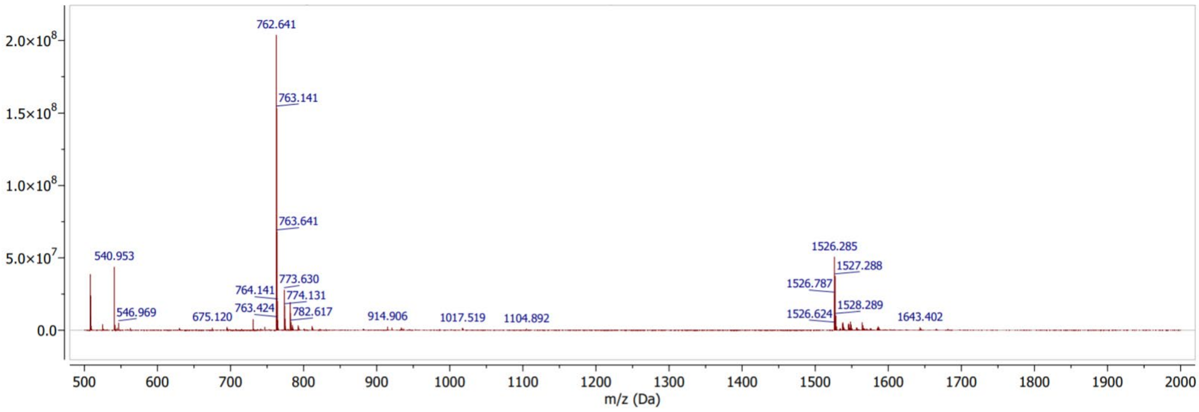

RNA 3 cleavage fragment 2 (2',3'-cyclic phosphate, 5'-AGAGUUC-G-AA-3') $C_{97}H_{118}N_{42}O_{68}P_{10}$
Monoisotopic mass calc. 3268.444, obs. 3268.498

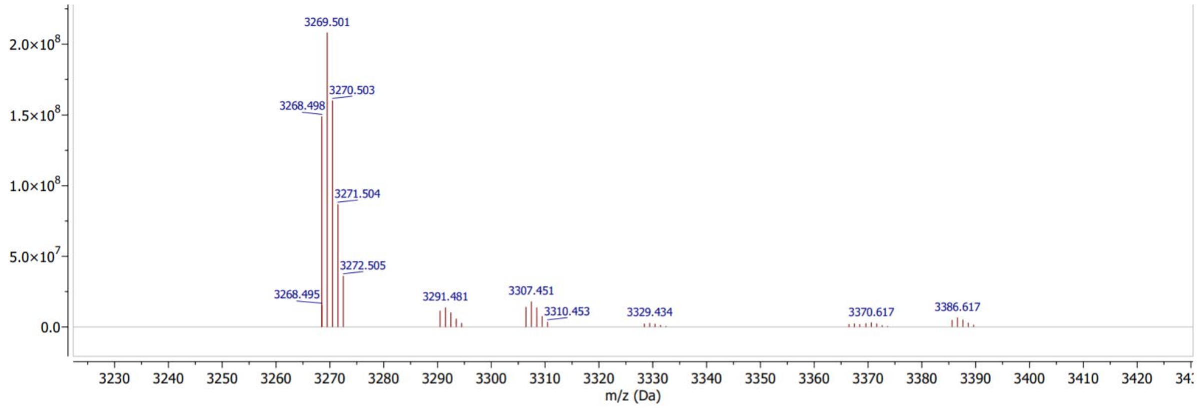

RNA 4 cleavage fragment 1 (5'-AGCCC-3') $C_{47}H_{61}N_{19}O_{32}P_4$
Monoisotopic mass calc. 1527.268, obs. 1527.295.

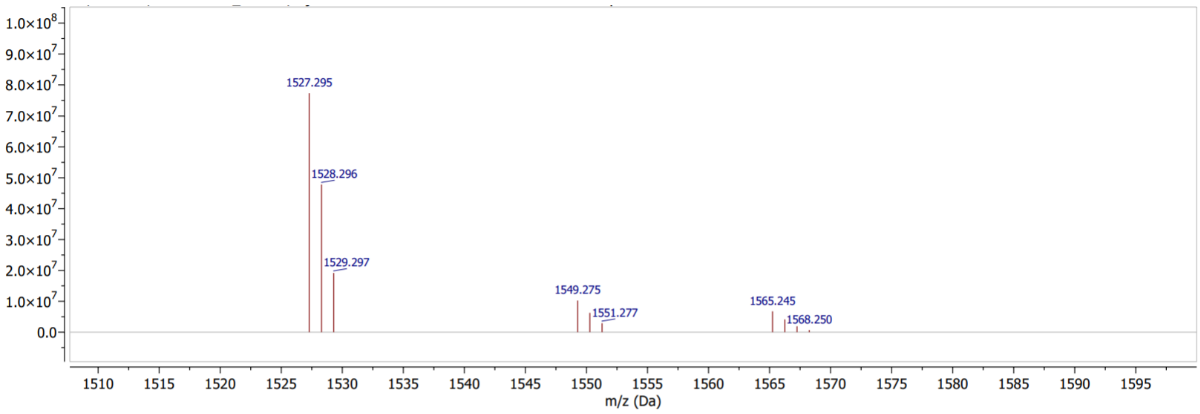

RNA 4 cleavage fragment 2 (2',3'-cyclic phosphate, 5'-AGAGUUC-A-AA-3') $C_{97}H_{118}N_{42}O_{67}P_{10}$
Monoisotopic mass calc. 3252.449, obs. 3252.507

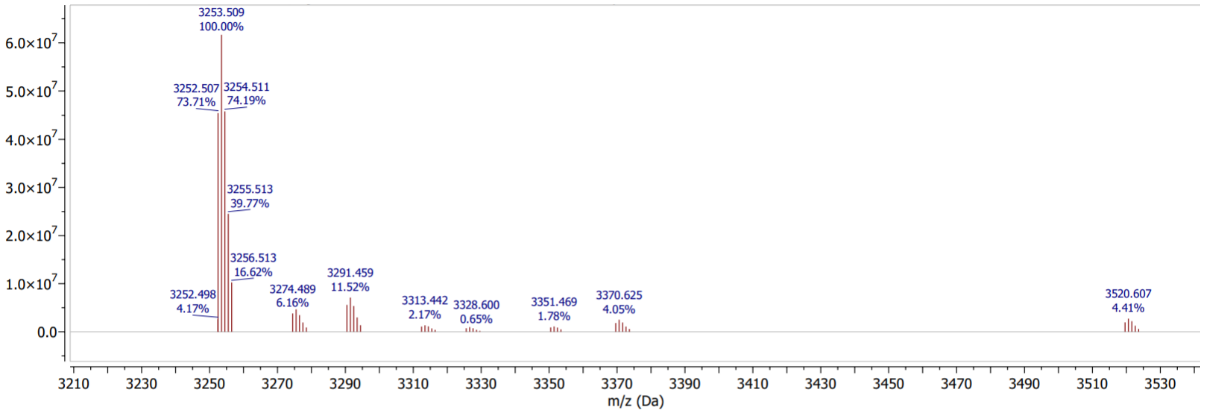

RNA 4 cleavage fragment 3 (2',3'-cyclic phosphate, 5'-AGAGUUC-A-AAA-3') $C_{107}H_{130}N_{47}O_{73}P_{11}$
Monoisotopic mass calc. 3581.502, obs. 3581.561

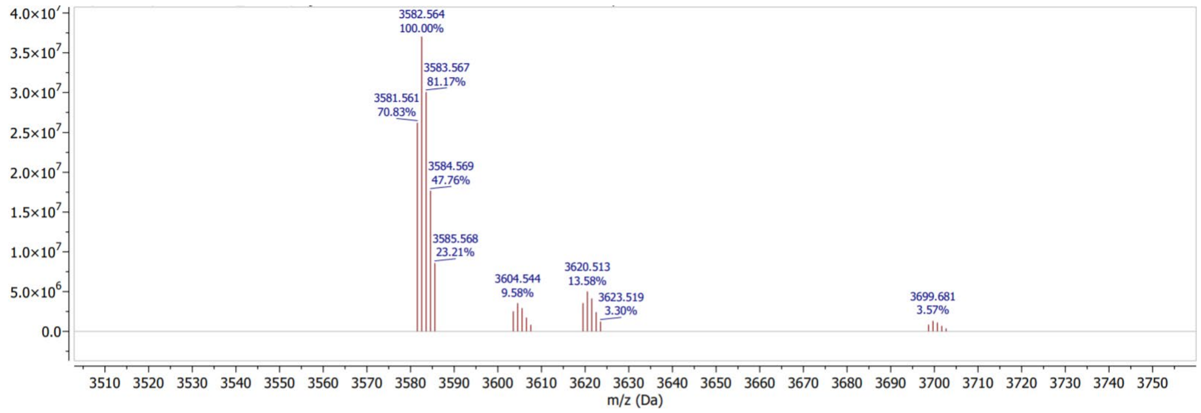

S-3.3 The extent of cleavage of 3-nucleotide bulge-forming RNAs 6-9 by *PNAzyme IV*

Timepoints at which the aliquots were quenched are shown on each chromatogram. These data are shown in Fig 3 in the main text.

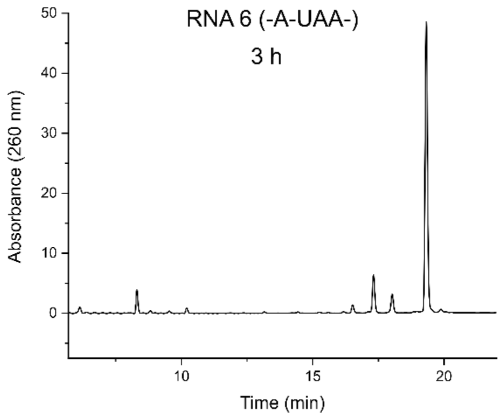

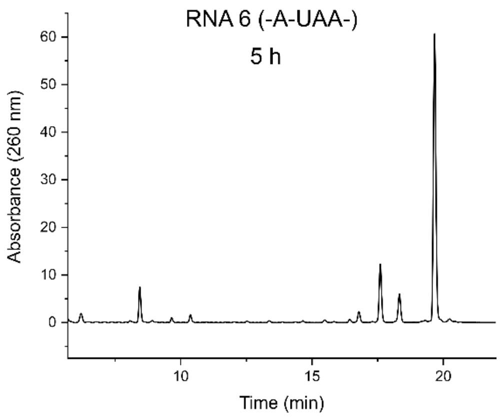

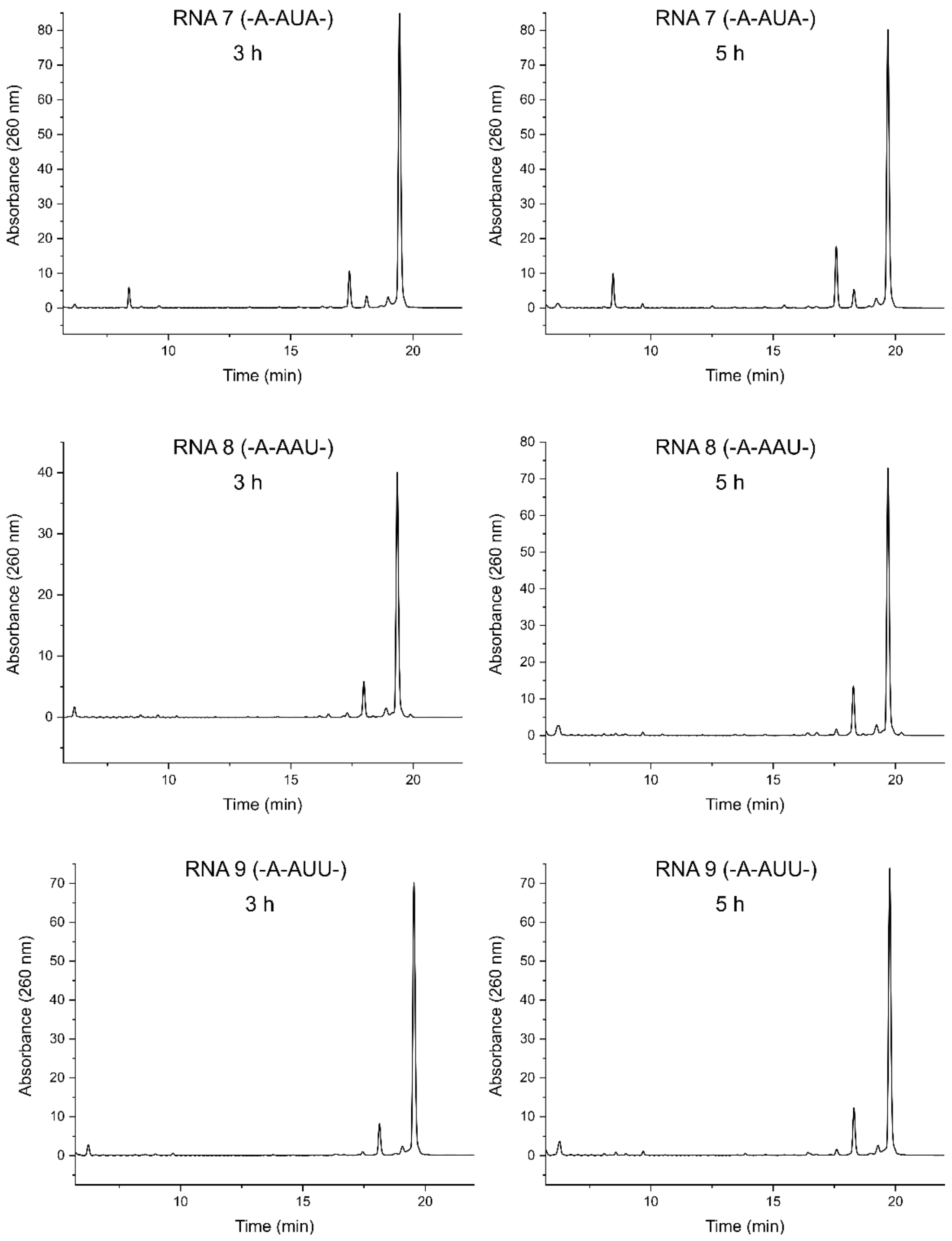
RNA 7 (-A-AUA-)
3 h
RNA 7 (-A-AUA-)
5 h
RNA 8 (-A-AAU-)
3 h
RNA 8 (-A-AAU-)
5 h
RNA 9 (-A-AUU-)
3 h
RNA 9 (-A-AUU-)
5 h
Absorbance (260 nm)
Time (min)

S-3.4. The extent of cleavage of 3-nucleotide bulge-forming RNAs 10-12 by *PNAzyme V*

Timepoints at which the aliquots were quenched are shown on each chromatogram. These data are shown in Fig 3 in the main text.

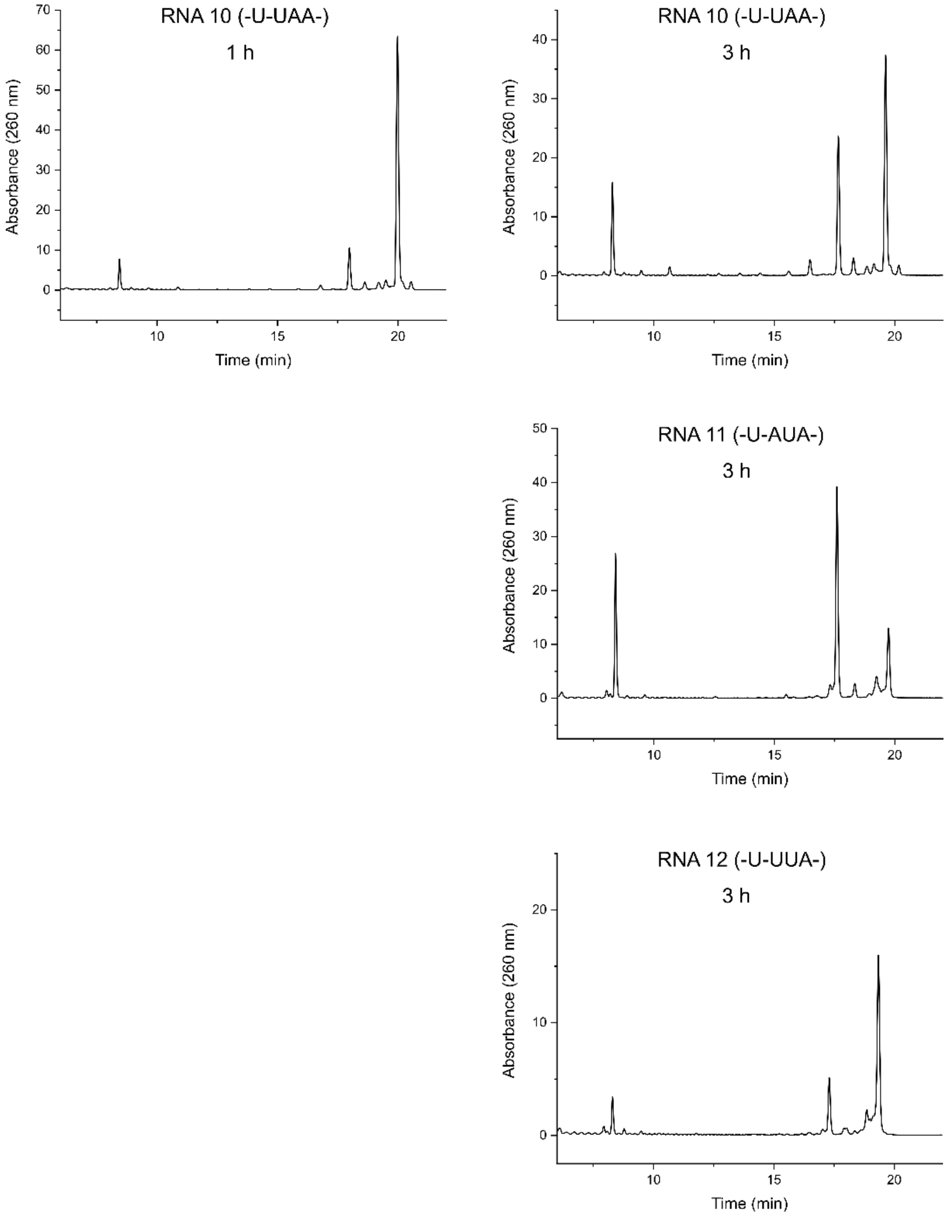

S-3.5. MS analysis of RNA fragments from the cleavage of RNA 11 by *PNAzyme V*

RNA 11 cleavage fragment 1 (5'-AGCCC-3') $C_{47}H_{61}N_{19}O_{32}P_4$
Monoisotopic mass calc. 1527.268, obs. 1527.299.

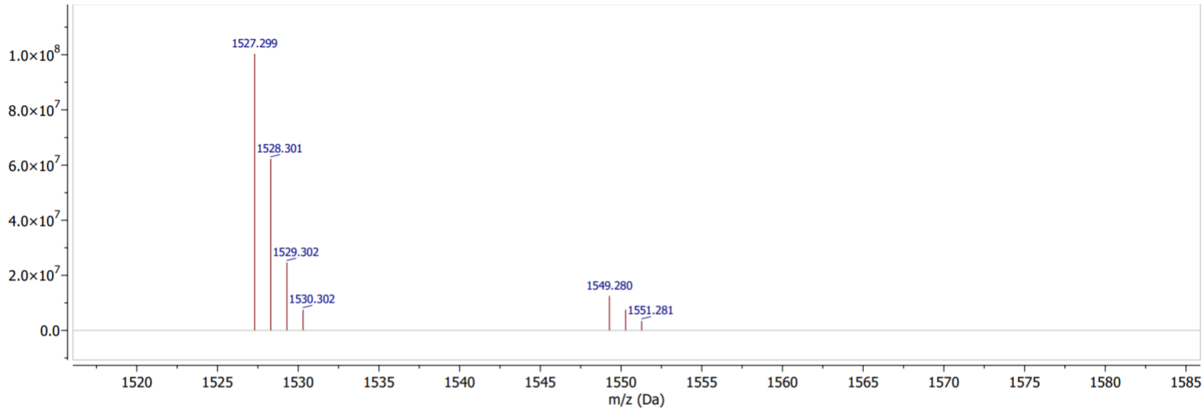

RNA 11 cleavage fragment 2 (2',3'-cyclic phosphate, 5'-AGAGUUC-U-AU-3') $C_{95}H_{116}N_{36}O_{71}P_{10}$
Monoisotopic mass calc. 3206.395 obs. 3206.461

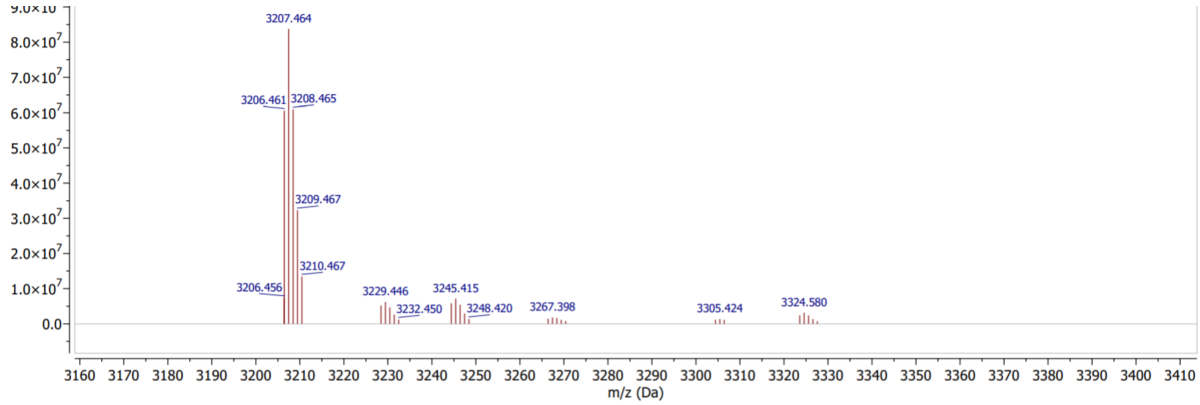

S-3.6. The extent of cleavage of 2-nucleotide bulge-forming RNAs 13-15 by *PNAzyme IV*

Timepoints at which the aliquots were quenched are shown on each chromatogram. These data are summarised in Fig 4 in the main text.

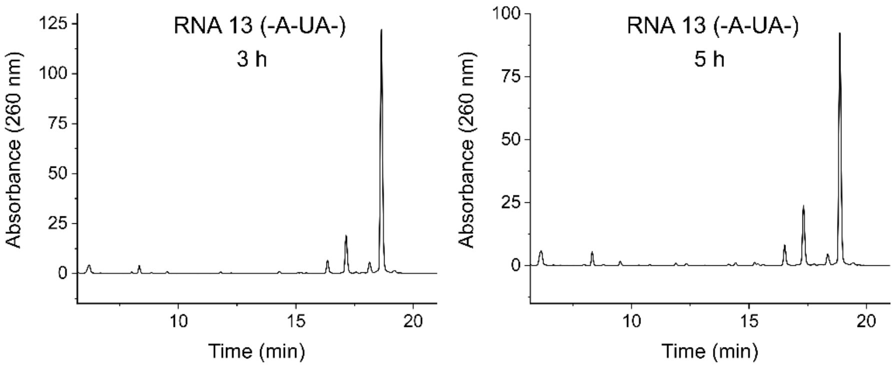

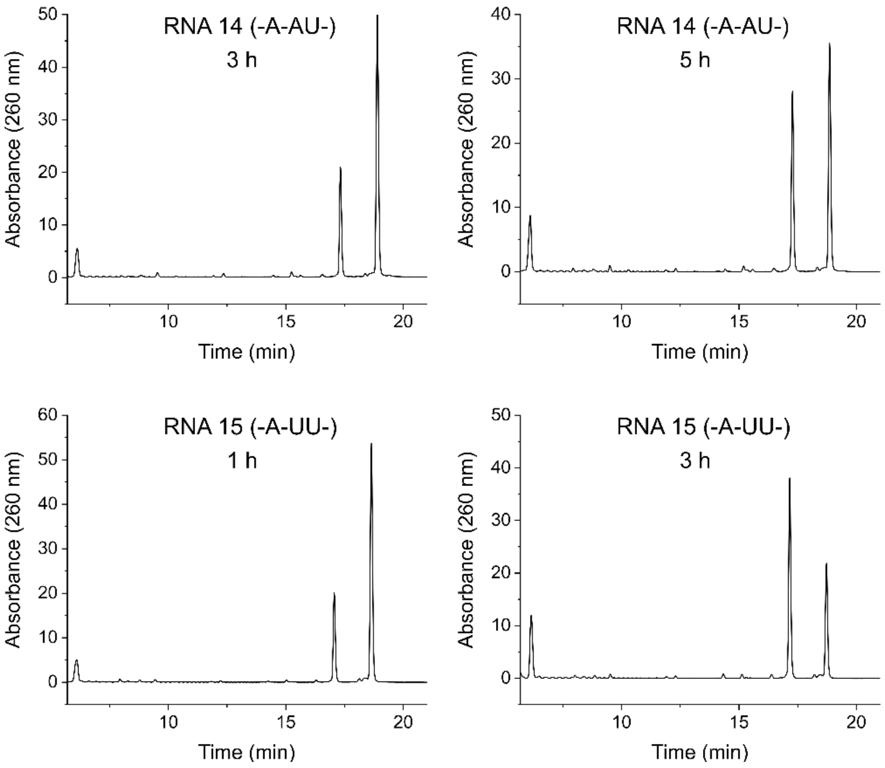

S-3.7. MS analysis of RNA fragments from the cleavage of RNAs 13, 15 by *PNAzyme IV*

RNA 13 cleavage fragment 1 (5'-AGCCC-3') $C_{47}H_{61}N_{19}O_{32}P_4$
Monoisotopic mass calc. 1527.268, obs. 1527.298.

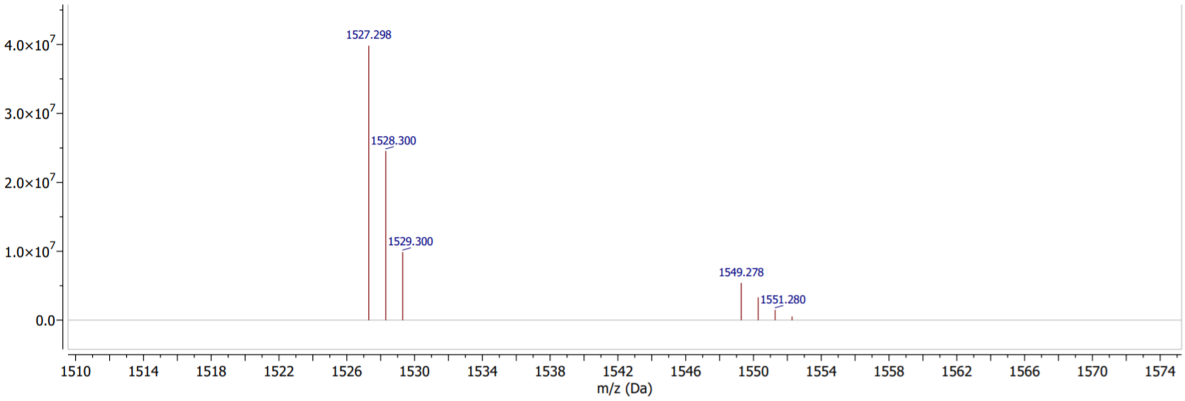

RNA 13 cleavage fragment 2 (2',3'-cyclic phosphate, 5'-AGAGUUC-A-U-3') $C_{86}H_{105}N_{34}O_{63}P_9$
Monoisotopic mass calc. 2900.370 obs. 2900.425

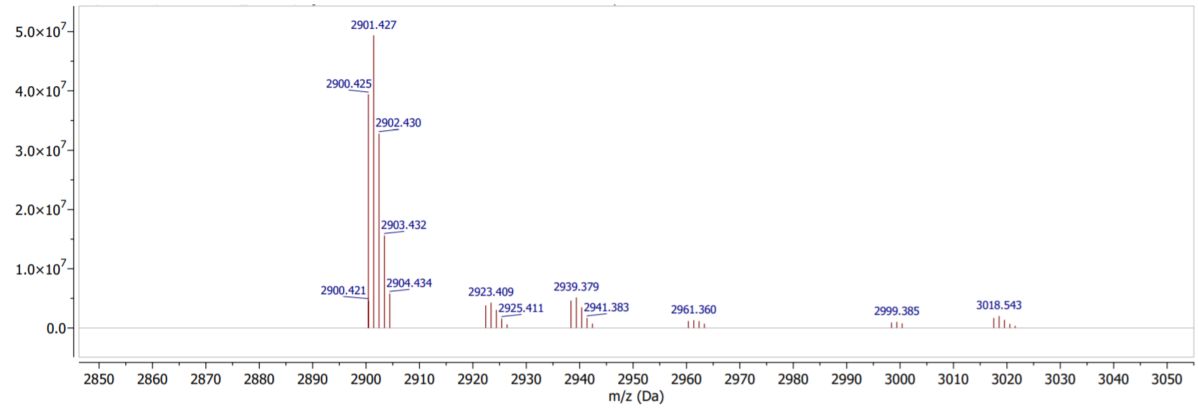

RNA 13 cleavage fragment 3 (2',3'-cyclic phosphate, 5'-AGAGUUC-A-UA-3') $C_{96}H_{117}N_{39}O_{69}P_{10}$
Monoisotopic mass calc. 3229.422 obs. 3229.488

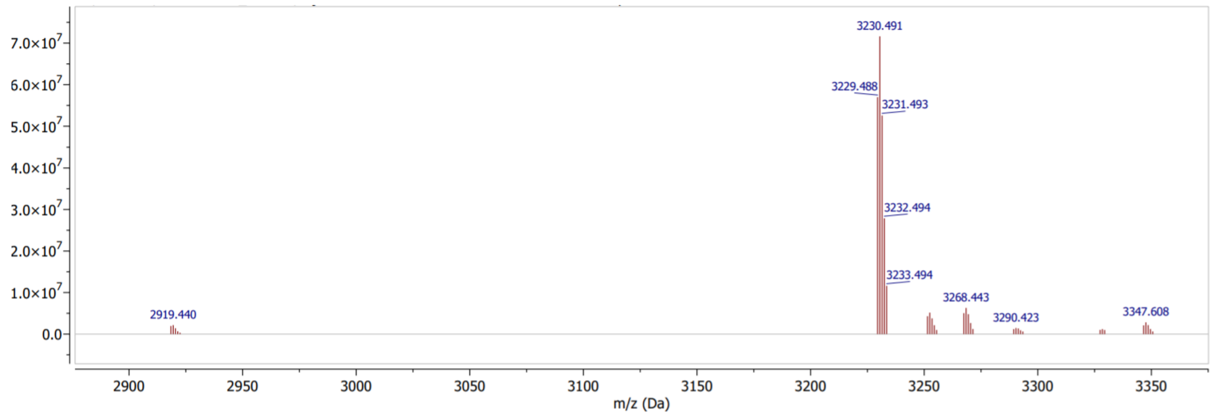

RNA 15 cleavage fragment 1 (2',3'-cyclic phosphate, 5'-AGA-GUUC-A-UU-3') $C_{95}H_{116}N_{36}O_{71}P_{10}$
Monoisotopic mass calc. 3206.395, obs. 3206.458.

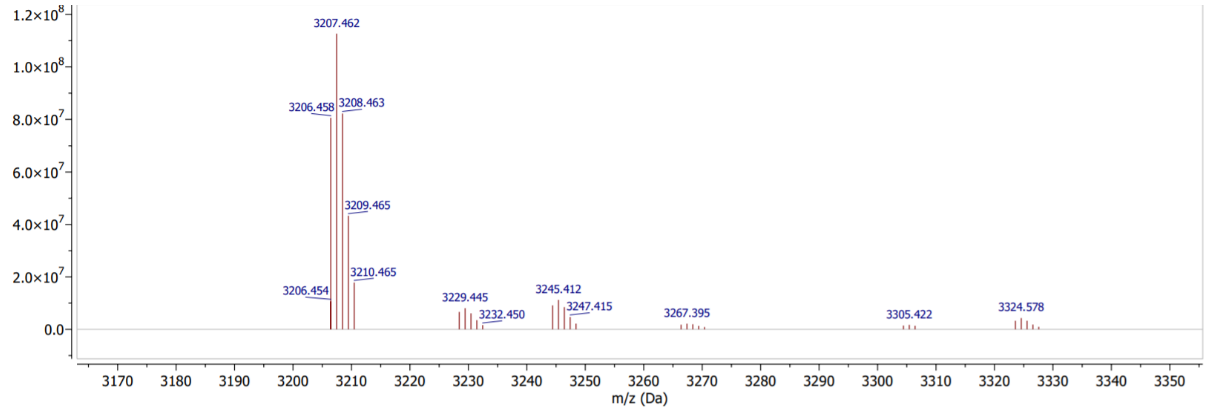

S-3.8. The extent of cleavage of 2-nucleotide bulge-forming RNAs 16-17 by *PNAzyme V*

Timepoints at which the aliquots were quenched are shown on each chromatogram. These data are summarised in Fig 4 in the main text.

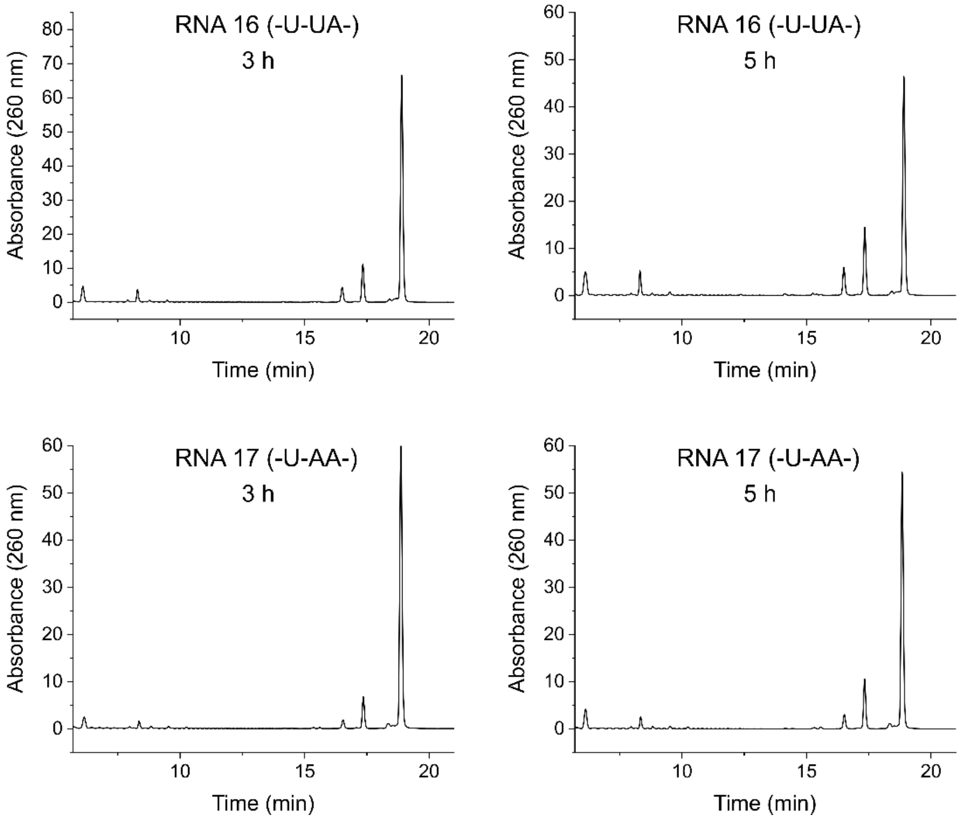

S-3.9. MS analysis of RNA fragments from the cleavage of RNA 17 by *PNAzyme V*

RNA 17 cleavage fragment 1 (5'-AGCCC-3') $C_{47}H_{61}N_{19}O_{32}P_4$
Monoisotopic mass calc. 1527.268, obs. 1527.298.

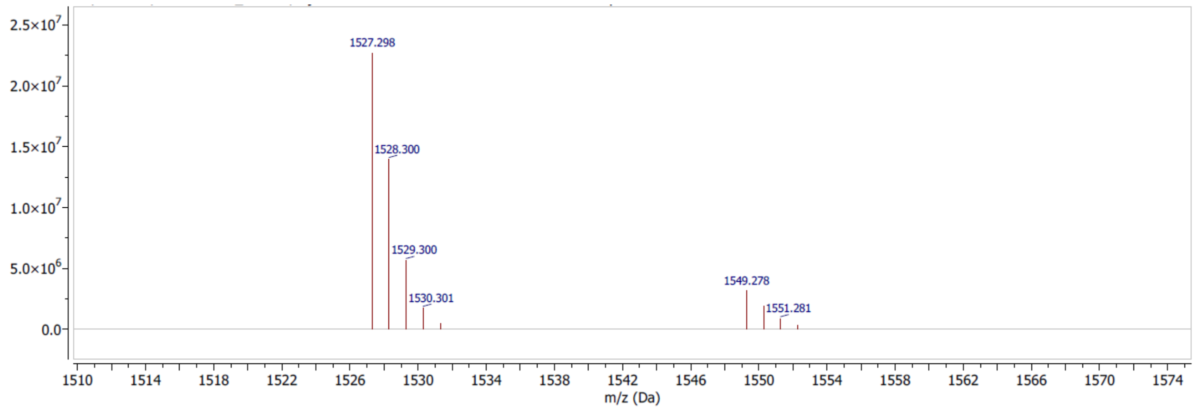

RNA 17 cleavage fragment 2 (2',3'-cyclic phosphate 5'-AGA-GUUC-U-A-3') $C_{86}H_{105}N_{34}O_{63}P_{9}$
Monoisotopic mass calc. 2900.370, obs. 2900.425.

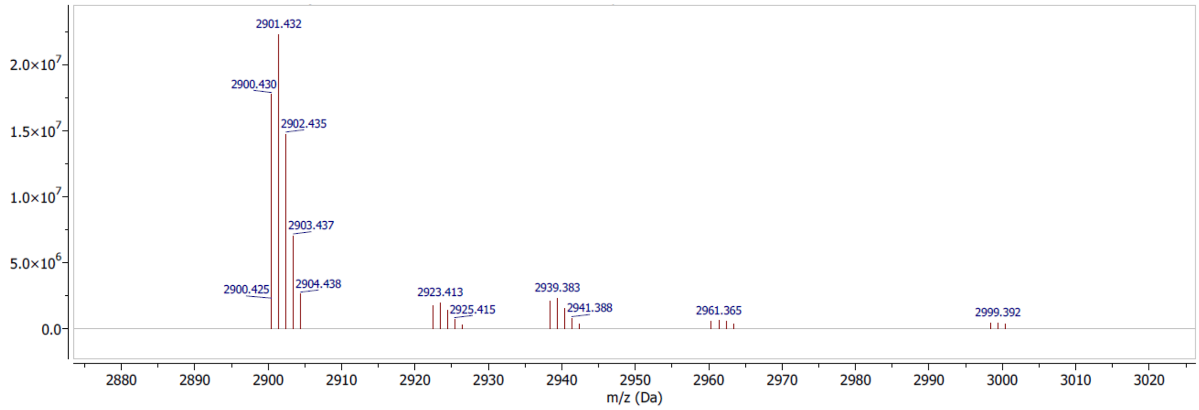

RNA 17 cleavage fragment 3 (2',3'-cyclic phosphate 5'-AGA-GUUC-U-AA-3') $C_{96}H_{117}N_{39}O_{69}P_{10}$
Monoisotopic mass calc. 3229.422, obs. 3229.480.

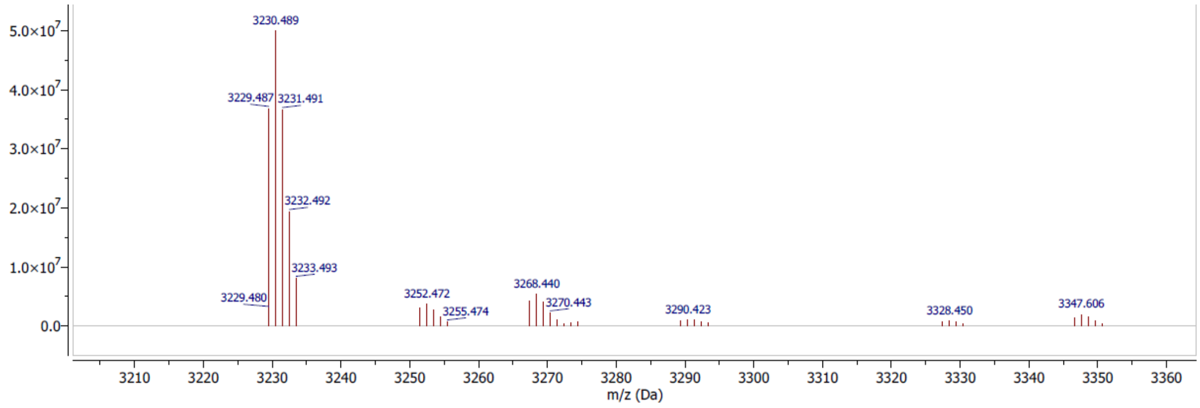

S-3.10 The extent of cleavage of UA bulges in RNAs 18-19 by *PNAzymes II-III*

Timepoints at which the aliquots were quenched are shown on each chromatogram. These data are summarised in Fig 4 in the main text.

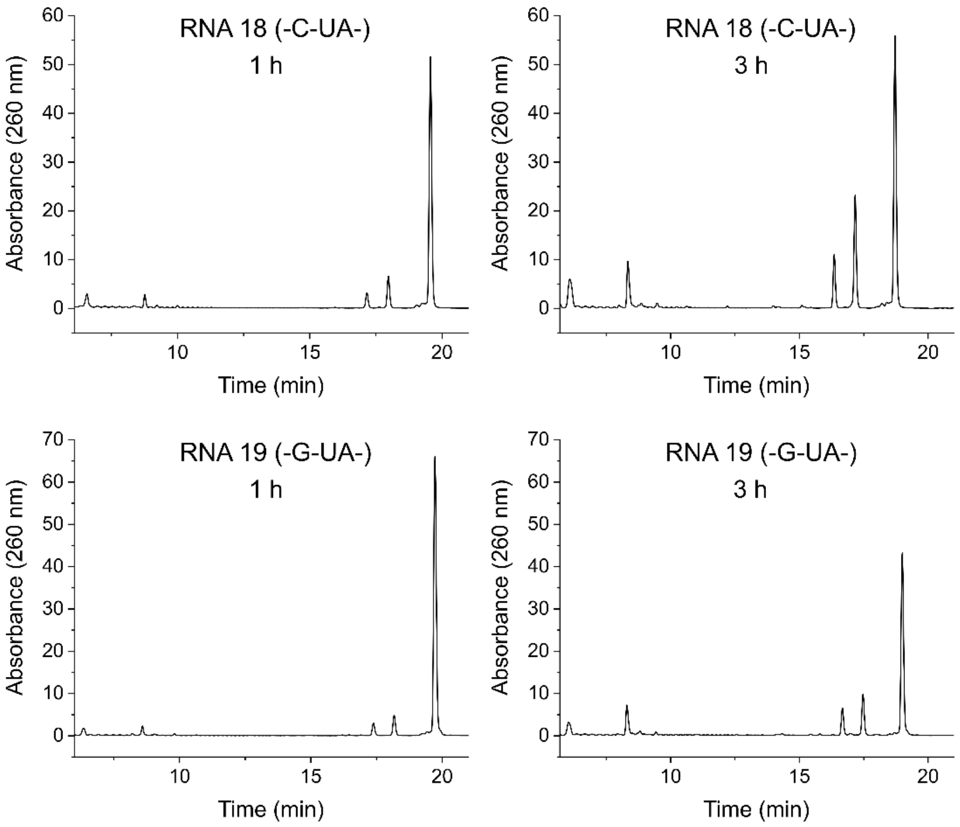

S-3.11. MS analysis of RNA fragments from the cleavage of RNA 18 by *PNAzyme II*

RNA 18 cleavage fragment 1 (5'-AGCCC-3') $C_{47}H_{61}N_{19}O_{32}P_4$
Monoisotopic mass calc. 1527.268, obs. 1526.285.

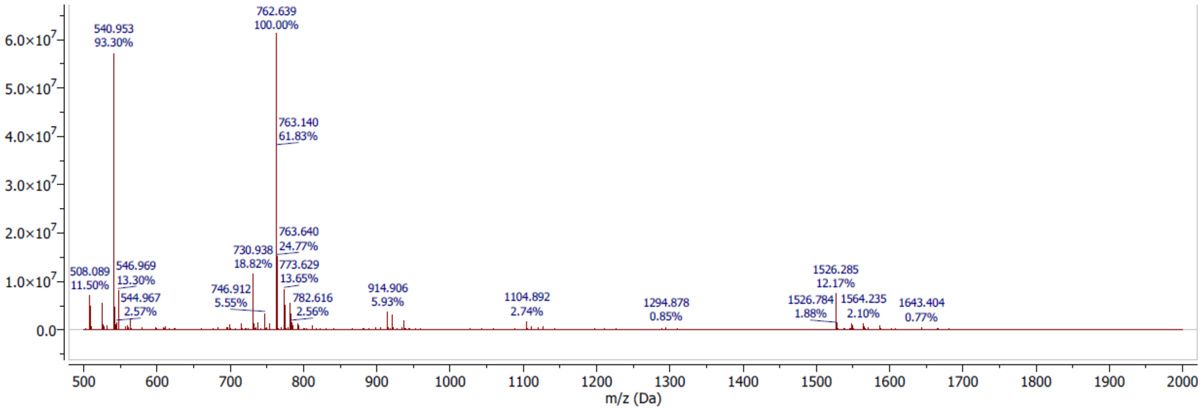

RNA 18 cleavage fragment 2 (2',3'-cyclic phosphate 5'-AGA-GUUC-C-U-3') $C_{85}H_{105}N_{32}O_{64}P_9$
Monoisotopic mass calc. 2876.358, obs. 2876.408.

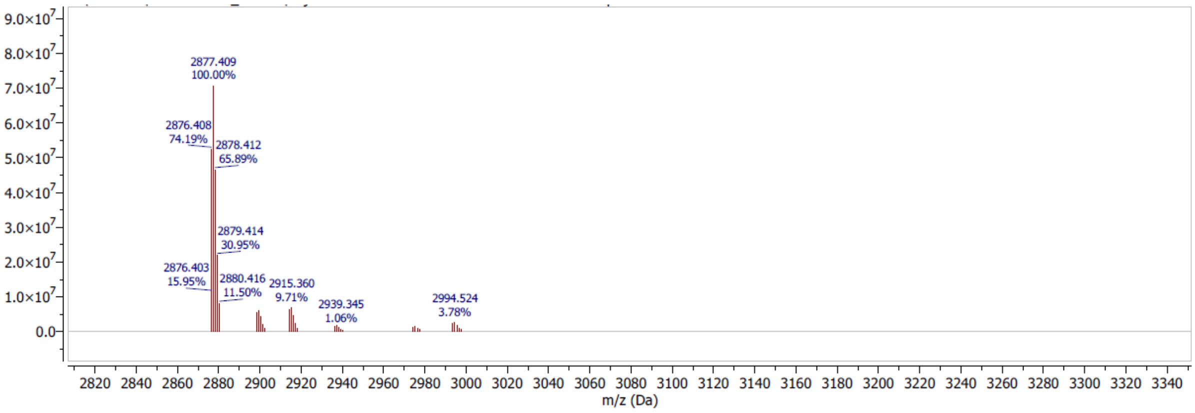

RNA 18 cleavage fragment 3 (2',3'-cyclic phosphate 5'-AGA-GUUC-C-UA-3') $C_{95}H_{117}N_{37}O_{70}P_{10}$
Monoisotopic mass calc. 3205.410, obs. 3205.461

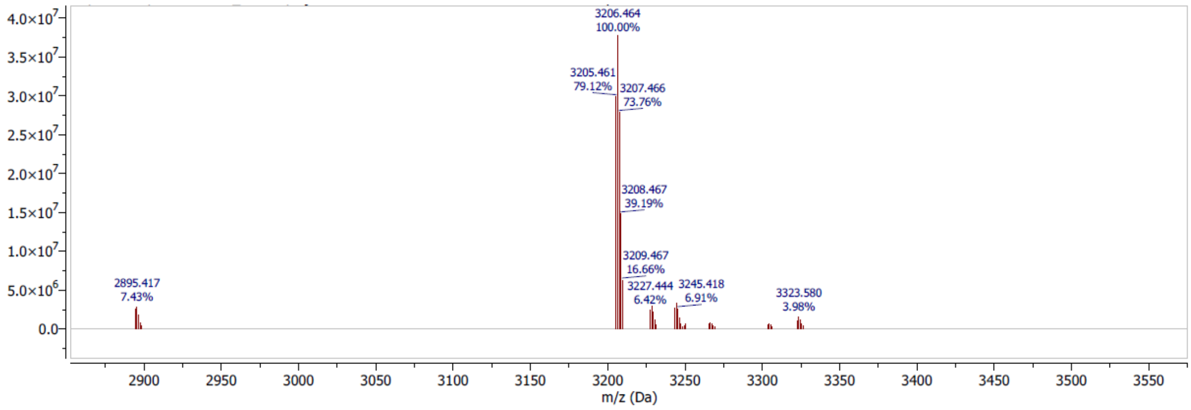

S-3.12. MS analysis of RNA fragments from the cleavage of RNA 19 by *PNAzyme III*

RNA 19 cleavage fragment 1 (5'-AGCCC-3') $C_{47}H_{61}N_{19}O_{32}P_4$
Monoisotopic mass calc. 1527.268, obs. 1526.285.

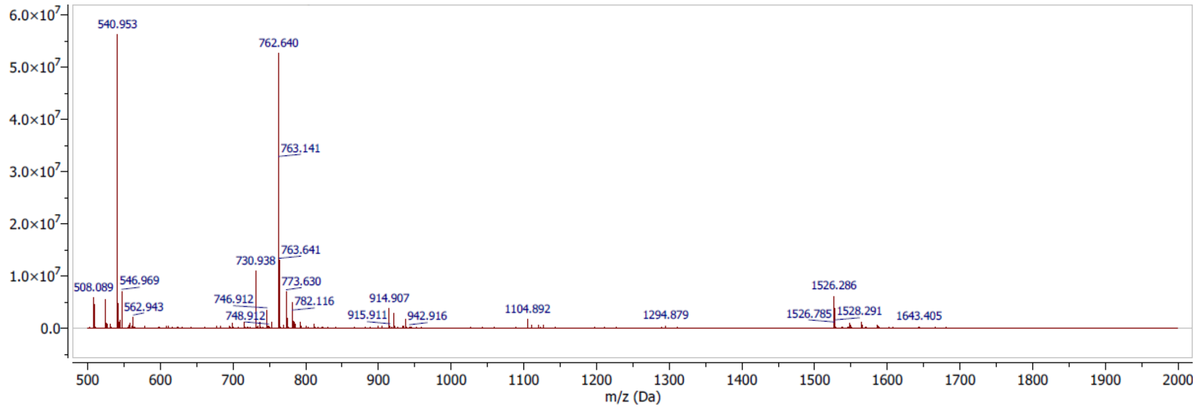

RNA 19 cleavage fragment 2 (2',3'-cyclic phosphate 5'-AGA-GUUC-G-U-3') $C_{86}H_{105}N_{34}O_{64}P_9$
Monoisotopic mass calc. 2916.365, obs. 2916.414.

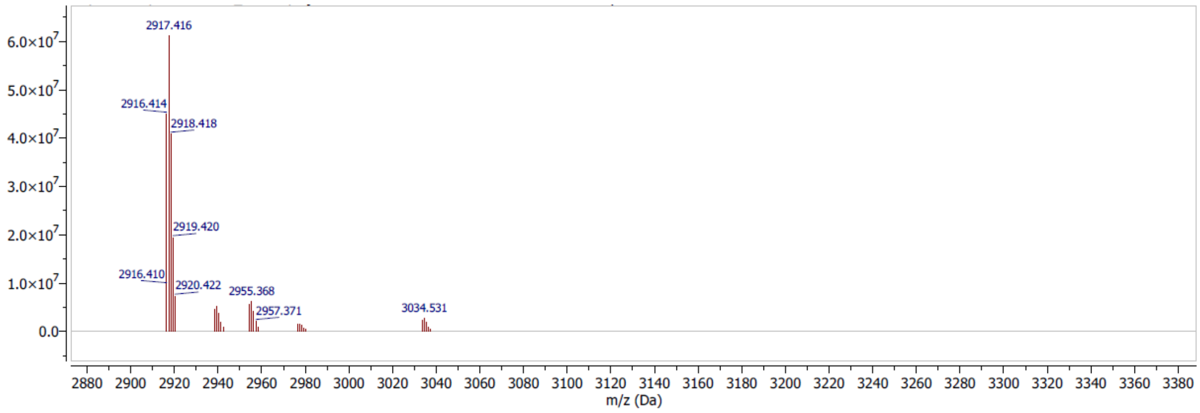

RNA 19 cleavage fragment 3 (2',3'-cyclic phosphate 5'-AGA-GUUC-G-UA-3') $C_{96}H_{117}N_{39}O_{70}P_{10}$
Monoisotopic mass calc. 3245.417, obs. 3245.469.

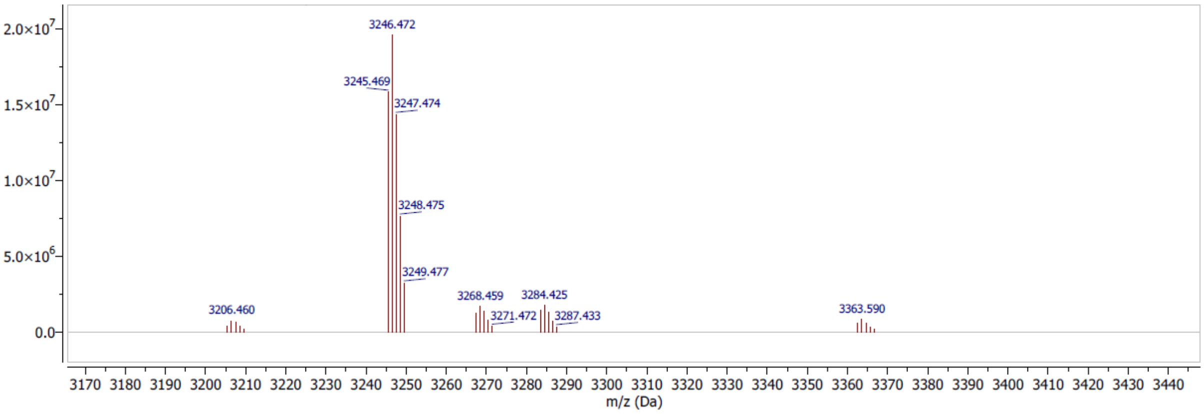

S-3.13 The extent of cleavage of RNA 6 by *PNAzyme VII*

Timepoints at which the aliquots were quenched are shown on each chromatogram. These data are summarised in Fig 5 in the main text.

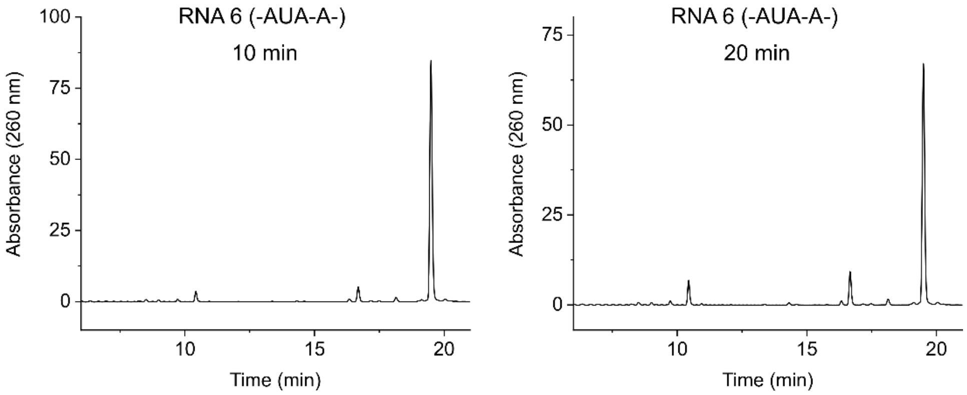

S-3.14. MS analysis of RNA fragments from the cleavage of RNA 6 by *PNAzyme VII*

RNA 6 cleavage fragment 1 (5'-A-AGCCC-3') $C_{57}H_{73}N_{24}O_{38}P_5$
Monoisotopic mass calc. 1856.321, obs. 1856.357.

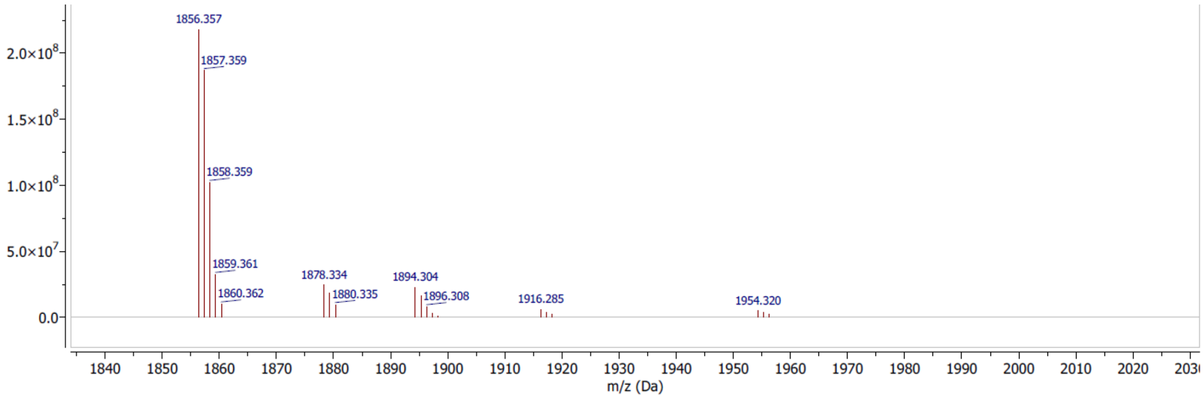

RNA 6 cleavage fragment 2 (2',3'-cyclic phosphate 5'-AGA-GUUC-AU-3') $C_{86}H_{105}N_{34}O_{63}P_9$
Monoisotopic mass calc. 2900.370, obs. 2900.424.

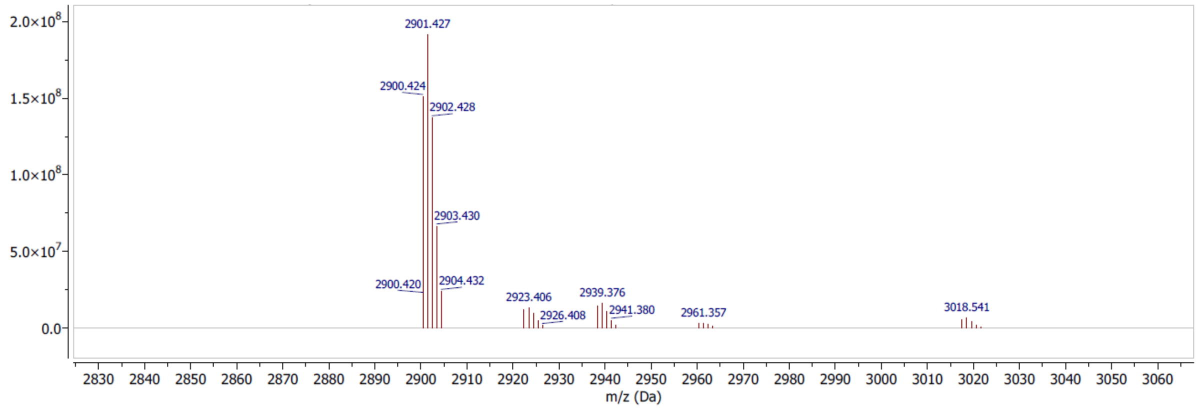

S-3.15 The extent of cleavage of 4-nucleotide bulge-forming RNAs 4-12 by *PNAzyme I*

Timepoints at which the aliquots were quenched are shown on each chromatogram. These data are summarised in Fig 8 in the main text. The chromatogram corresponding to the cleavage of RNA 4 by *PNAzyme I* in 3 h has been reported previously.[1]

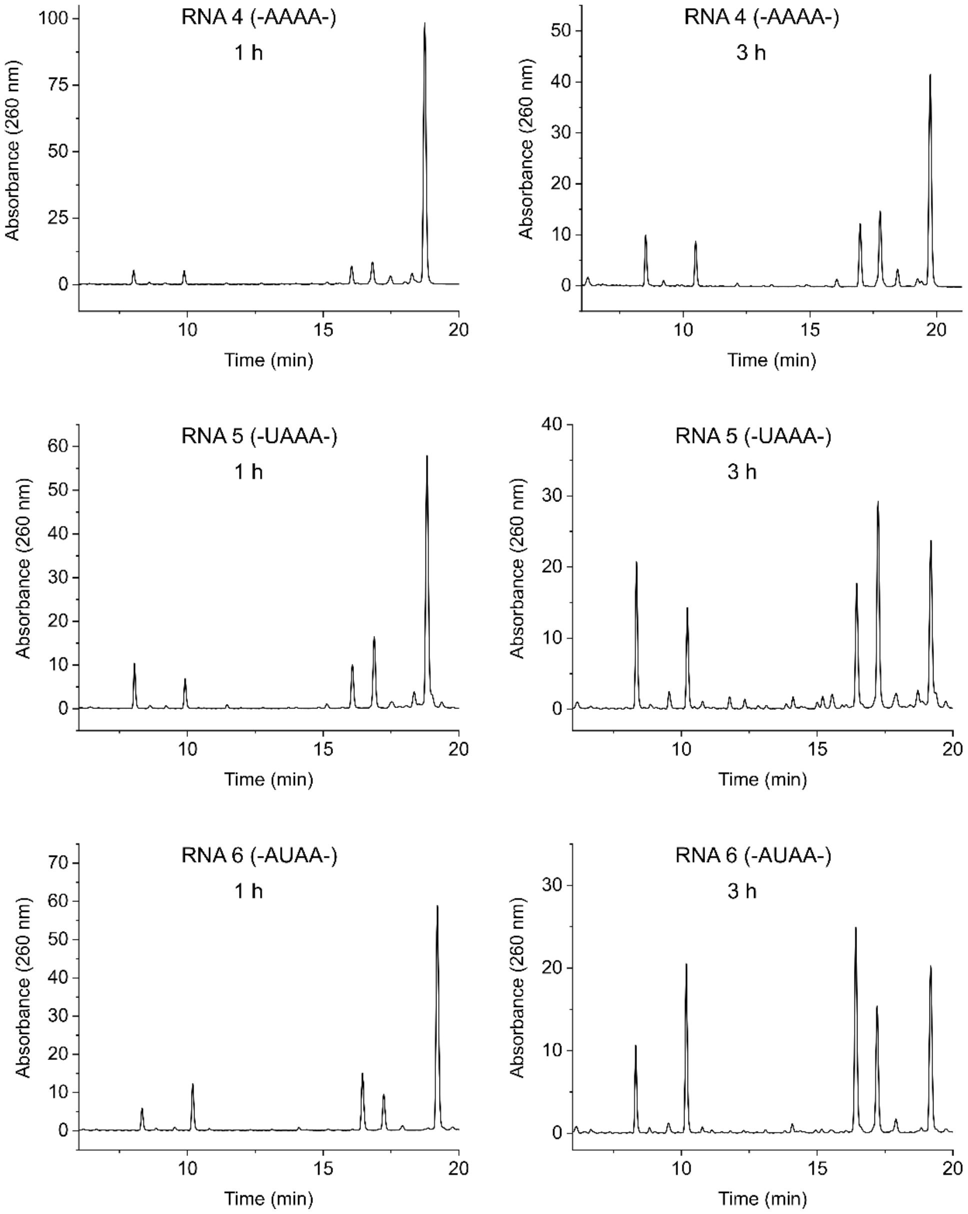

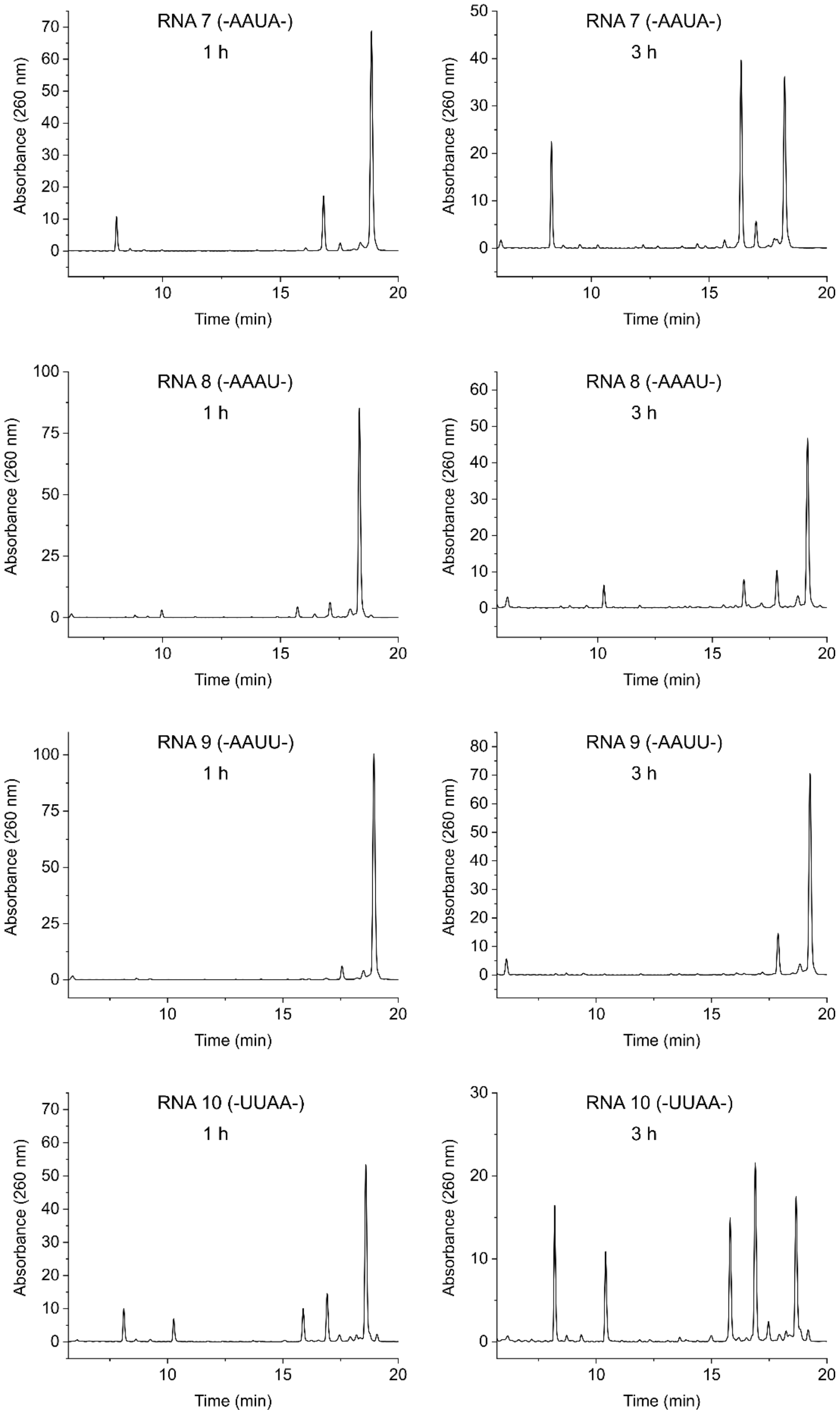
RNA 7 (-AAUA-)
1 h
RNA 7 (-AAUA-)
3 h
RNA 8 (-AAAU-)
1 h
RNA 8 (-AAAU-)
3 h
RNA 9 (-AAUU-)
1 h
RNA 9 (-AAUU-)
3 h
RNA 10 (-UUAA-)
1 h
RNA 10 (-UUAA-)
3 h
Absorbance (260 nm)
Time (min)

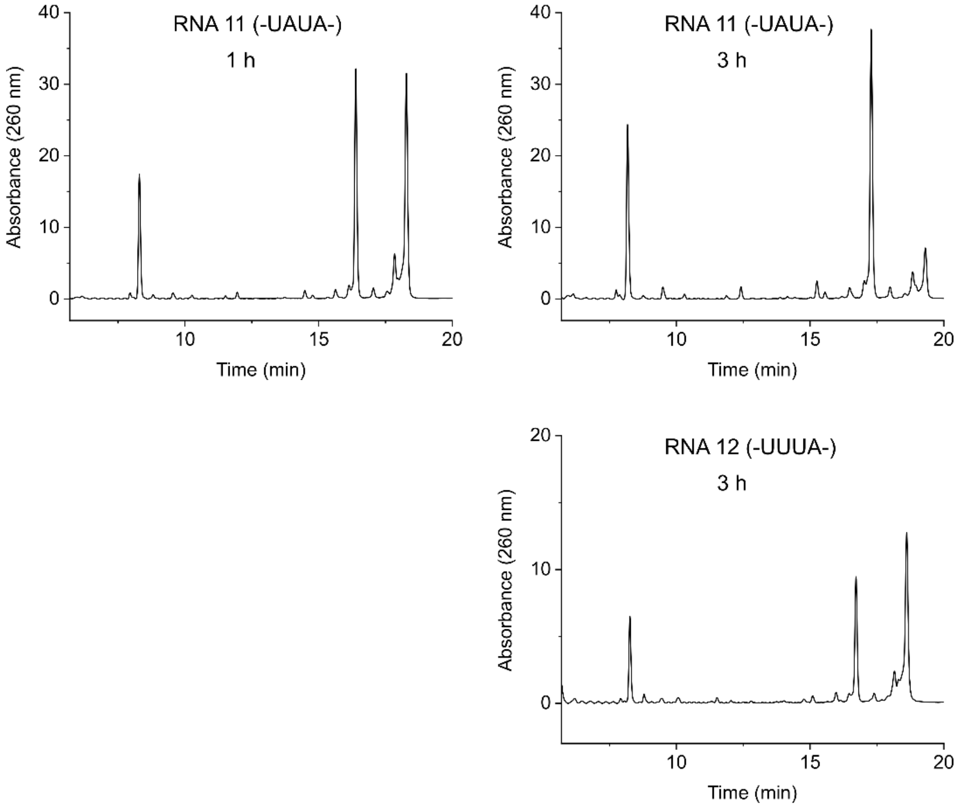

S-3.16. MS analysis of RNA fragments from the cleavage of RNAs 4 and 11 by *PNAzyme I*

RNA 4 cleavage fragment 1 (5'-AGCCC-3') $C_{47}H_{61}N_{19}O_{32}P_4$
Monoisotopic mass calc. 1527.268, obs. 1527.292.

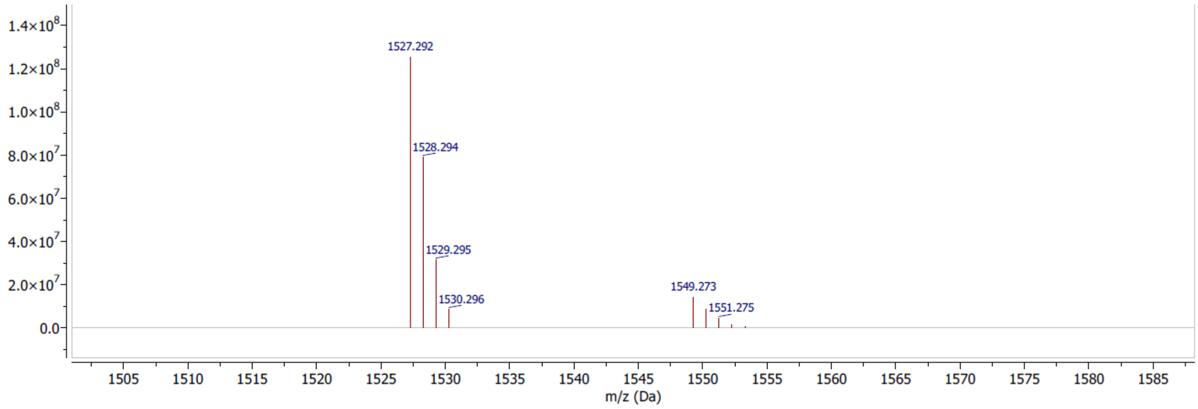

RNA 4 cleavage fragment 2 (5'-AA-GCCC-3') $C_{57}H_{73}N_{24}O_{38}P_5$
Monoisotopic mass calc. 1856.321, obs. 1856.351.

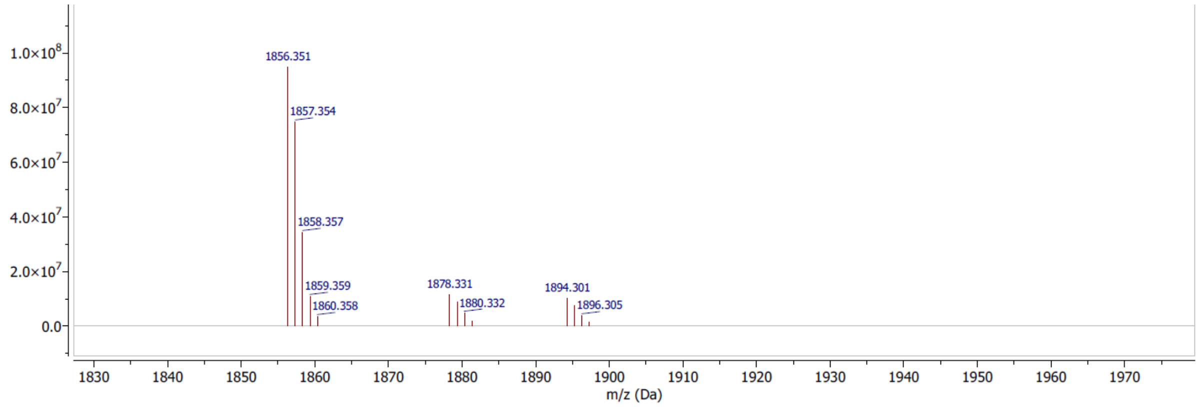

RNA 4 cleavage fragment 3 (2',3'-cyclic phosphate, 5'-AGAGUUC-AA-3') $C_{87}H_{106}N_{37}O_{61}P_9$
Monoisotopic mass calc. 2923.397, obs. 2923.445.

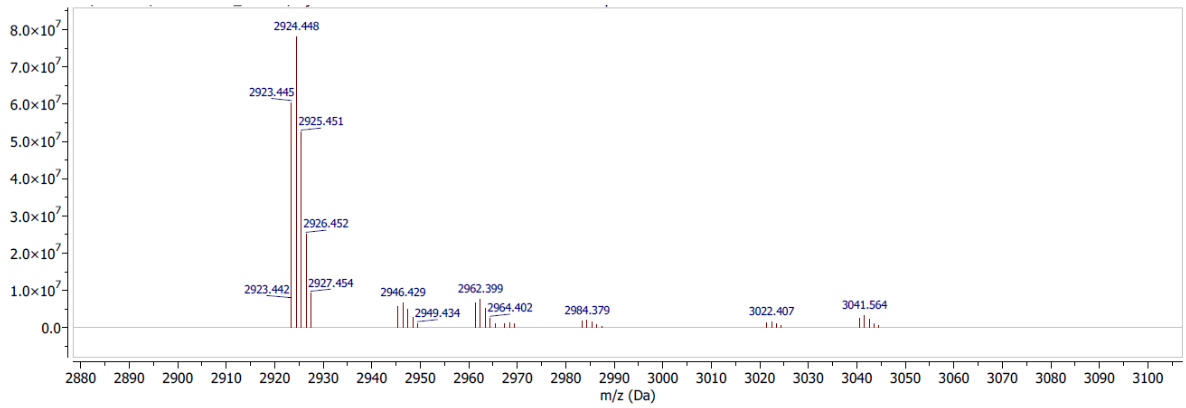

RNA 4 cleavage fragment 4 (2',3'-cyclic phosphate, 5'-AGAGUUC-AAA-3') $C_{97}H_{118}N_{42}O_{67}P_{10}$
Monoisotopic mass calc. 3252.449, obs. 3252.501

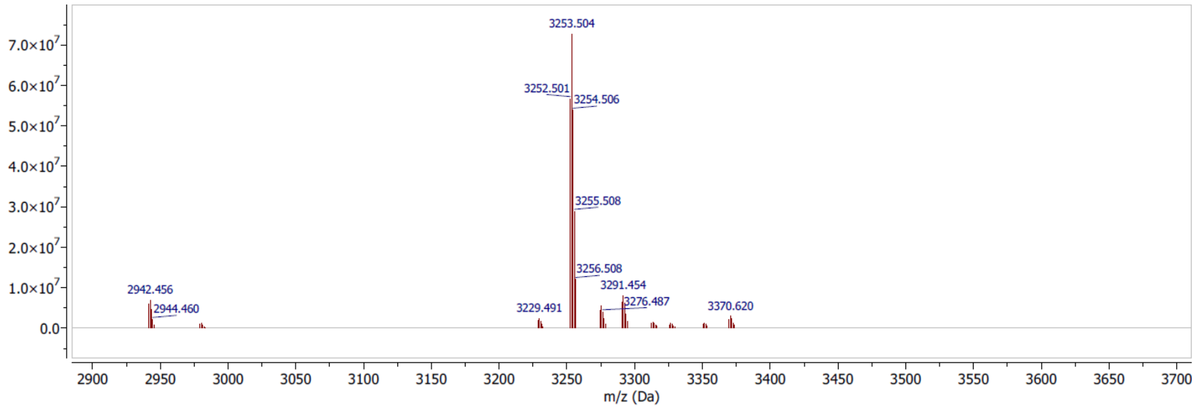

RNA 11 cleavage fragment 1 (5'-AGCCC-3') $C_{47}H_{61}N_{19}O_{32}P_4$
Monoisotopic mass calc. 1527.268, obs. 1526.285

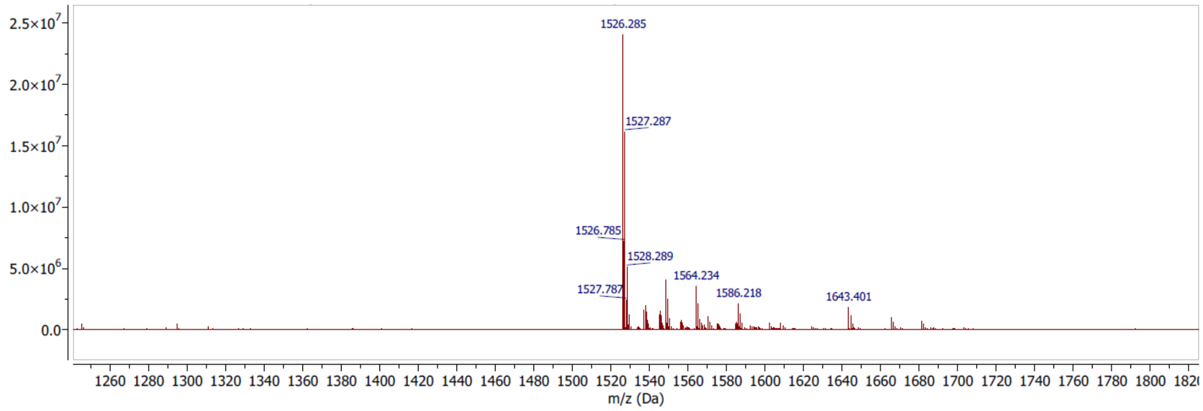

RNA 11 cleavage fragment 2 (2',3'-cyclic phosphate, 5'-AGAGUUC-UAU-3') $C_{95}H_{116}N_{36}O_{71}P_{10}$
Monoisotopic mass calc. 3206.395, obs. 3206.447

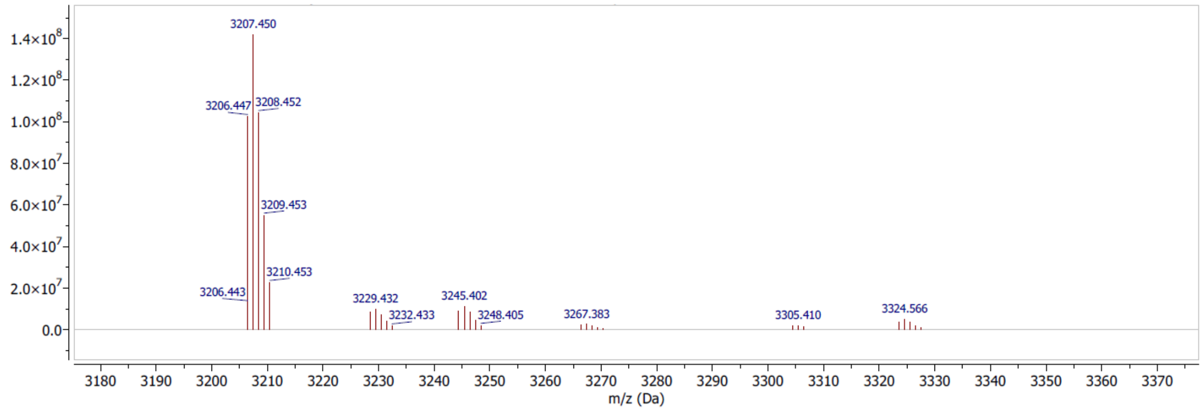

S-4. Circular Dichroism Spectroscopy of RNA/PNAzyme complexes

S-4.1. Complexes where the bulge-closing base pair varies in the long recognition arm

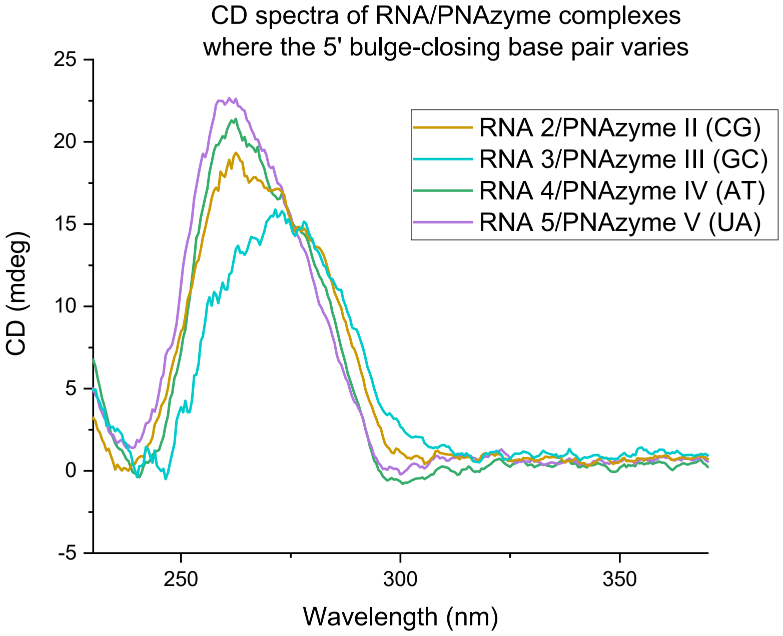

S-4.2 Complexes with a GT wobble vs a GC Watson-Crick base pair closing the bulge in the short recognition arm

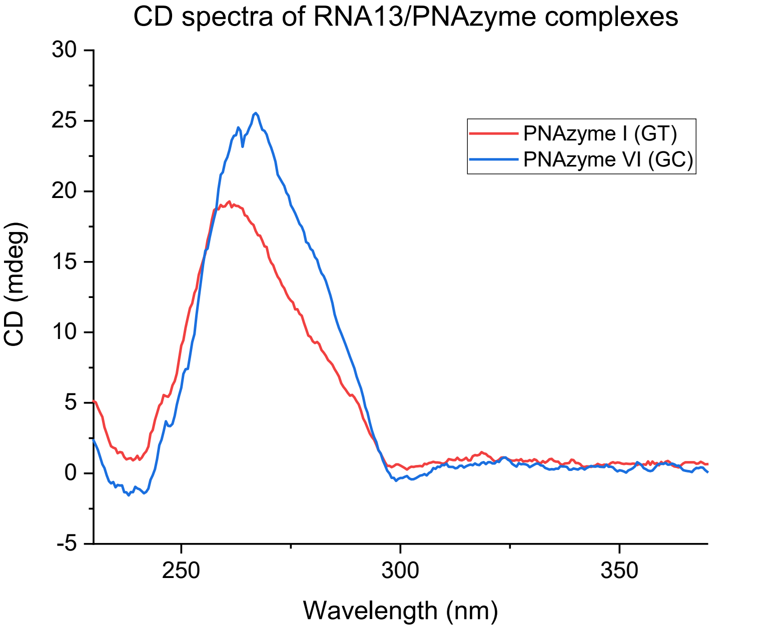

S-5. RNA cleavage experiments in the presence of Mg^{2+} and K^+

Extent of cleavage of RNA 13 (AUA bulge) by PNAzyme I at pH 7.0

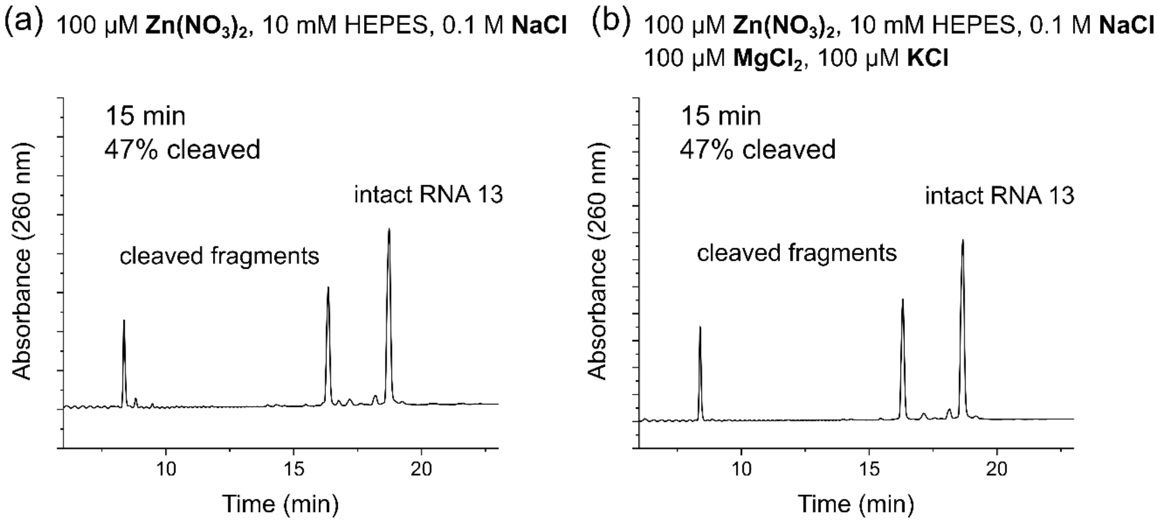

* (a) has been previously reported by Luige et al[1]

References

1 O. Luige, P. P. Bose, R. Stulz, P. Steunenberg, O. Brun, S. Andersson, M. Murtola and R. Strömberg, *Chem. Commun.*, 2021, **57**, 10911–10914.
